# SMILES-based QSAR and molecular docking studies of chalcone analogues as potential anti-colon cancer

**DOI:** 10.1038/s41598-025-91338-9

**Published:** 2025-02-24

**Authors:** Abolfazl Askarzade, Shahin Ahmadi, Ali Almasirad

**Affiliations:** 1 Department of Medicinal Chemistry, Faculty of Pharmacy, Tehran Medical Sciences, Islamic Azad University, Tehran, Iran; 2 Department of Pharmaceutical Chemistry, Faculty of Pharmaceutical Chemistry, Tehran Medical Sciences, Islamic Azad University, Tehran, Iran

**Keywords:** QSAR, Molecular docking, Chalcone derivatives, HT-29, CORAL, Biochemistry, Cancer, Computational biology and bioinformatics, Drug discovery, Diseases

## Abstract

QSAR modeling was applied to predict the anti-colon activity (against HT-29) of 193 chalcone derivatives using the Monte Carlo method, based on the index of ideality correlation (IIC) target function. The models were constructed using CORAL software, which employed optimal descriptors combining SMILES notation and hydrogen-suppressed molecular graphs (HSG). Among the developed models, Split #2 was identified as the best-performing model, with R^2^_validation = 0.90, IIC_validation = 0.81, and Q^2^_validation = 0.89. The mechanistic interpretation of the models, utilizing enhancing/reducing promoters, demonstrated that the models are capable of accurately predicting the pIC_50_ values of other chalcone derivatives with high robustness and precision. Based on these promoters, ten new compounds were selected from the ChEMBL database for pIC_50_ prediction, and molecular docking was performed using the protein with PDB ID:1SA0.

**Supplementary Information:**

The online version contains supplementary material available at 10.1038/s41598-025-91338-9.

## Introduction

Colon cancer is recognized as one of the most common cancers worldwide^1,2^ and is the fourth leading cause of cancer-related deaths globally^2^. Notably, colon cancer affects both men and women at nearly equal rates. Studies have shown significant variations in the incidence of colorectal cancer among different ethnic groups. For instance, the prevalence is higher in the U.S. and Europe compared to Asian countries such as Japan, which may be attributed to differences in dietary habits^1^.

Studies have indicated that a well-balanced diet rich in vegetables, fruits, and flavonoids may reduce the risk of cancer, with flavonoids specifically known for their anticancer properties^3^. Recent research has shown that combination therapies using cytotoxic agents such as 5-fluorouracil, oxaliplatin, and leucovorin, along with surgical intervention, can improve survival rates in patients with advanced colon cancer^4–6^. Despite the efficacy of these treatments, scientists continue to search for new compounds that HT-29 cells do not develop resistance to. These novel agents may offer more targeted effects with fewer side effects.

**Fig. 1:**
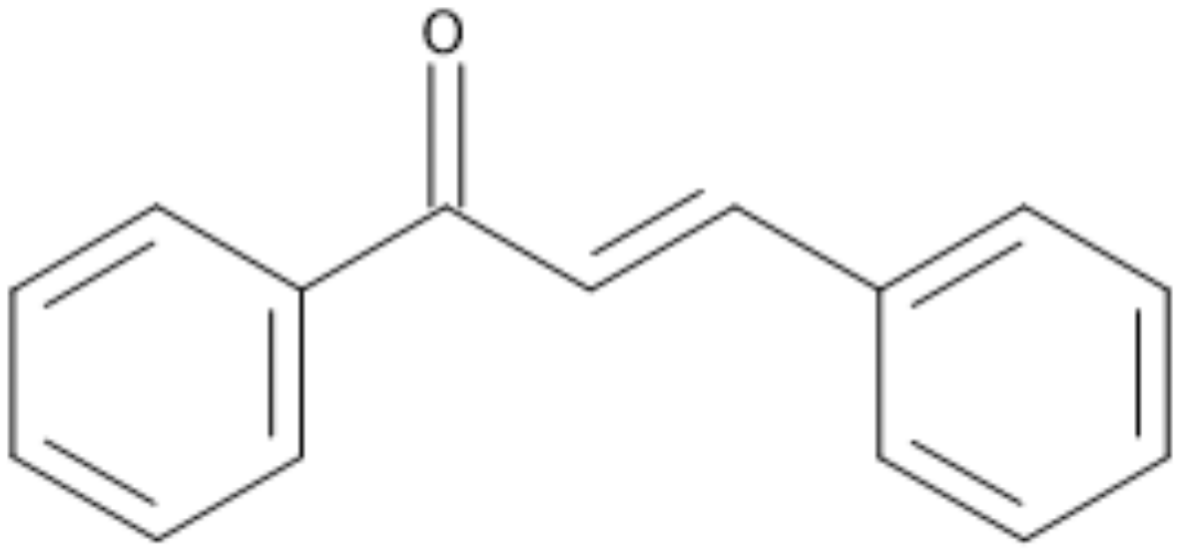
Chalcones chemical structure.

Flavonoids are a diverse group of plant-derived compounds, known as secondary metabolites. Subcategories of flavonoids include isoflavones, flavones, flavonols, anthocyanins, and chalcones, all of which exhibit health-promoting properties, such as anticancer, anti-inflammatory, and antioxidant activities^7,8^. Chalcones (Fig. 1) are chemical compounds within the flavonoid family. They are scientifically classified as 1,3-diphenylprop-2-en-1-one, consisting of two aromatic rings linked by an unsaturated three-carbon bridge. The term ‘chalcone’ is derived from the Greek word ‘chalcos,’ referring to the bronze color characteristic of many chalcone compounds in nature^9^. Chalcone scaffolds are commonly found in vegetables, fruits, berries, tea, and other plants. Notably, chalcones exhibit significant anti-inflammatory and anticancer potential^10^. Studies have shown that natural chalcones and their derivatives are potent cytotoxic agents that can affect cancer cells, though their precise mechanisms of action remain poorly understood^11^.

Elafibranor, a prescription medication used for the treatment of primary biliary cholangitis (PBC), is an approved drug containing a chalcone structure^12^. Despite numerous reports highlighting the anti-cancer effects of chalcone derivatives in various cancers including colon cancer^13–16^, through mechanisms such as tubulin inhibition^14,17,18^, no clinical antitumor drugs currently feature this structure. As a result, chalcone derivatives have attracted significant interest and are anticipated to contribute to advancements in antitumor drug development.

QSAR (Quantitative Structure-Activity Relationship) models are methods designed to establish a well-defined correlation between the chemical structure of a compound and its biological activity. QSAR models can reveal how specific structural features either enhance or diminish biological activity^19^. Additionally, QSAR is recognized as a valuable tool in drug design and development^20^.

In the 21st century, medicinal chemistry research has increasingly concentrated on both natural and synthetic chalcones due to their wide-ranging pharmacological potential^21^. These molecules exhibit a variety of biological activities, including antibacterial^22^, antioxidant^23^, anticancer^24^, antiviral^25^, antidiabetic^26^, and acetylcholinesterase^27^ inhibitory effects, as well as functioning as non-purine xanthine oxidase^28^ inhibitors. Chalcones are particularly appealing for research because of their straightforward structure, ease of synthesis, and significant biological applications^21^.

The Monte Carlo optimization approach has recently gained attention as a structure-independent method for QSPR/QSAR modeling^29^. This method, utilizing optimal descriptors based on molecular graphs and SMILES, offers advantages over traditional descriptors. Moreover, combining Monte Carlo modeling with molecular docking studies demonstrates strong correlations in predicting and designing target-specific drugs. This approach enables the identification and design of compounds with desired biological activities^30^.

In this study, we aimed to develop a reliable QSAR model to predict the biological activities of chalcone derivatives using CORAL software, a free tool for QSAR modeling. CORAL utilizes the Monte Carlo method to determine descriptors of correlation weights (DCWs)^31–35^. The Monte Carlo method is a randomization process that helps classify data in an unbiased manner, improving the precision and reliability of QSAR modeling^36^. Recent research has focused on the anticancer potential of chalcones and their derivatives, with reports indicating significant activity against cancer cells. For example, in 2014, Rybka et al. investigated 162 chalcone molecules using seven molecular descriptors to develop a predictive QSAR model for evaluating their activity against HT-29 adenocarcinoma cell lines^37^. This model demonstrated excellent predictive capability based on the selected descriptors.

This research aims to establish a reliable and predictive QSAR model to elucidate the relationship between the structures of chalcone derivatives and their biological activities. The model will also demonstrate how descriptors of correlation weights (DCWs) influence the biological activity of these compounds. Additionally, we will use molecular docking to analyze several new compounds from the ChEMBL database, determining their binding interactions with the receptor and the strength of these interactions.

## Data set and methods

### Data set

In this QSAR study, 193 chalcone derivatives were collected from three papers that investigated the inhibitory activity of chalcones and their derivatives against HT-29 human colon adenocarcinoma cell lines^37–39^. The dependent variable used to evaluate the activity of each structure in these papers was IC_50_ (the concentration of a substance that inhibits a process by 50%). All compounds included in this QSAR modeling study were assessed for IC_50_ activity using the MTT assay, which measures the mitochondrial reduction of yellow tetrazolium dye (MTT) to blue formazan^40^. The dataset was selected based on the following criteria: high-quality experimental data, structural diversity of chalcone derivatives, relevance to HT-29 cell line activity, and sufficient size to support robust QSAR model development. This careful curation ensures that the dataset is reliable and applicable for predicting the activity of new chalcone derivatives against HT-29. IC_50_ values were converted to molar units and represented on a negative logarithmic scale as pIC_50_ (-logIC_50_). For the QSAR model, pIC_50_ values were used as the dependent variable, with a range from 3.58 to 7.00.

The structures of all chalcone derivative compounds are displayed in Table S1. These structures were drawn using BIOVIA Draw 2019 and then converted to SMILES notation for modeling with CORAL software. This dataset had not previously been used for QSAR modeling to evaluate chalcone inhibition against HT-29 cells. Using CORAL, four distinct data splits were developed: Training set (≈ 27%), Invisible training set (≈ 27%), Calibration set (≈ 23%), and Validation set (≈ 23%). Table S2 shows the chalcone IDs, SMILES notation, and DCWs (descriptor of correlation weights), along with experimental and predicted pIC_50_ values.

### Optimal descriptor

CORAL software offers three types of descriptors for the modeling process: SMILES-based, graph-based, and hybrid descriptors, which combine SMILES and molecular graphs. According to available literature, hybrid descriptors generally provide more accurate and higher-quality statistical models compared to using either SMILES-based^29^ or graph-based descriptors alone^41–43^.

In this study, to achieve the best statistical outcomes, both SMILES and HSG (hydrogen-suppressed graph) descriptors were utilized to construct the QSAR model. The following equation illustrates the QSAR model used to predict pIC_50_ of chalcone derivatives against HT-29 cell lines:

1 $${\text{p}\text{I}\text{C}}_{50}={\text{C}}_{0}+{\text{C}}_{1}\times\text{D}\text{C}\text{W}\left({\text{T}}^{\text{*}},{\text{N}}^{\text{*}}\right)$$

C0 represents the regression coefficient, while C1 denotes the slope, both calculated using the least-squares method. Descriptors of Correlation Weights (DCWs) are the optimal descriptors essential for modeling, derived from molecular features obtained from HSG and SMILES notation. In the Monte Carlo optimization method, T* represents the threshold value, and N* denotes the number of epochs for each cycle of the optimization process^35^.

Therefore, the hybrid optimal descriptor used to predict the pIC50 of chalcone derivatives is computed using the following equation:

2 $${}^{\text{H}\text{y}\text{b}\text{r}\text{i}\text{d}}\text{D}\text{C}\text{W}\left({\text{T}}^{\text{*}},{\text{N}}^{\text{*}}\right)={}^{\text{S}\text{M}\text{I}\text{L}\text{E}\text{S}}\text{D}\text{C}\text{W}\left({\text{T}}^{\text{*}},{\text{N}}^{\text{*}}\right)+{}^{\text{G}\text{r}\text{a}\text{p}\text{h}}\text{D}\text{C}\text{W}\left({\text{T}}^{\text{*}},{\text{N}}^{\text{*}}\right)$$

To obtain the optimal SMILES-based and graph-based descriptors using CORAL software, the following two equations are applied:

3 $$\begin{aligned} {}_{{}}^{{{\text{HSG}}}} {\text{DCW}}\left( {{\text{T}},{\text{~N}}_{{{\text{epoch}}}} } \right) = & {\text{CW~}}\left( {{\text{C}}5} \right) + {\text{CW~}}\left( {{\text{C}}6} \right) + \sum CW\left( {e0_{k} } \right) + \sum CW\left( {e1_{k} } \right) \\ & + \sum CW\left( {e0_{k} + e1_{k} } \right) + \sum CW\left( {\left| {e0_{k} - e1_{k} } \right|} \right) + \sum CW\left( {pt2_{k} } \right) \\ & + \sum CW\left( {pt3_{k} } \right) + \sum CW\left( {pt2_{k} + pt3_{k} } \right) + \sum CW(\left| {pt2_{k} - pt3_{k} } \right|) \\ & + \sum CW\left( {S2_{K} } \right) + \sum CW\left( {S3_{K} } \right) \\ & + \sum CW\left( {S2_{k} + S3_{k} } \right) + \sum CW(\left| {S2_{k} - S3_{k} } \right|) + \sum CW(nn_{k} ) \\ \end{aligned}$$

4 $$\begin{aligned} ^{{{\text{SMILES}}}} {\text{DCW}}\left( {{\text{T}},{\text{~N}}_{{{\text{epoch}}}} } \right) = & {\text{CW~}}\left( {{\text{BOND}}} \right) + {\text{CW~}}\left( {{\text{NOSP}}} \right) + {\text{CW~}}\left( {{\text{HARD}}} \right) \\ & + \sum CW\left( {{\text{HALO}}} \right) + \sum CW\left( {{\text{PAIR}}} \right) + \sum CW\left( {S_{k} } \right) \\ & + \sum CW\left( {SS_{k} } \right) + \sum CW\left( {SSS_{k} } \right) \\ & + CW\left( {C_{{max}} } \right) + CW\left( {N_{{max}} } \right) + CW\left( {O_{{max}} } \right) + CW\left( {S_{{max}} } \right) \\ \end{aligned}$$

The detailed interpretation of each graph invariant and SMILES attribute in Eqs. (3) and (4) is provided in previously reported papers^44–48^. In these equations, CW(Z) represents the correlation weight of a parameter, which could be either SMILES-based or graph-based. Z refers to parameters such as C5, C6, e0k, e1k, p2k, p3k, S2k, S3k, nnk, BOND, NOSP, HARD, HALO, PAIR, Sk, SSk, SSSk, Cmax, Nmax, Omax, and Smax. Definitions of SMILES attributes and graph invariants are displayed in Table 1.

**Table 1:** The definitions of SMILES attributes and graph invariants.

	ID	Definition
SMILES attribute	Sk	Fragments of SMILES containing one symbol (e.g. ‘C’, ‘N’, ‘=’, ‘(‘ etc.) or a group of symbols that cannot be examined separately (e.g., ‘Cl’, ‘Br’, Si’, etc.)
	SSk	Fragments of SMILES containing two symbols (e.g. ‘cc’, ‘C(‘, etc.)
	SSSk	Fragments of SMILES containing three symbols (e.g. ‘CCC’, ‘c(c’, ‘C = C’, etc.)
	BOND	Presence or absence of chemical bonds: double (=), triple (#), and stereochemical (@) or @@).
	PAIR	Association two of BOND, NOSP, and HALO
	HARD	Association of BOND, NOSP, and HALO in the united structural code
	NOSP	Presence or absence of different chemical elements: nitrogen (N), oxygen (O), sulfur (S), and phosphorus (P);
	C_max_	Maximum number of rings
	N_max_	Maximum number of nitrogen atoms in a molecule
	O_max_	Maximum number of oxygen atoms in a molecule structure
Graph invariant	e2k	Morgan extended connectivity of first order
	e3k	Morgan extended connectivity of second-order
	pt2k	Number of paths of lengths 2 starting from a given vertex in the graph
	pt3k	Number of paths of length 3 starting from a given vertex in the graph
	S2_k_	Valence shells of the second orders
	S3_k_	Valence shells of the third orders
	C5 and C6	Codes of rings (five-member and six-member rings, with the data on the presence or absence of heteroatoms, aromaticity, and the total number of given rings in the molecule)

CORAL software can establish a QSAR model using two different approaches: (1) balancing correlation without applying the Index of Ideality of Correlation (IIC), referred to as TF1 (Target Function 1), and (2) balancing correlation with the application of IIC, referred to as TF2^49,50^. TF1 and TF2 are computed using the following Eq. 

5 $${\text{T}\text{F}}_{1}={\text{R}}_{\text{T}\text{R}\text{N}}+{\text{R}}_{\text{i}\text{T}\text{R}\text{N}}-\left|{\text{R}}_{\text{T}\text{R}\text{N}}-{\text{R}}_{\text{i}\text{T}\text{R}\text{N}}\right|\times\text{c}$$

6 $${\text{T}\text{F}}_{2}={\text{T}\text{F}}_{1}+\text{I}\text{I}\text{C}\times\text{c}$$

where RTRN and RiTRN represent the correlation coefficients for the training set and the invisible training set, respectively. The empirical constant (c) is typically used consistently^51^.

The Index of Ideality of Correlation (IIC) is calculated using data from the calibration set.

7 $$\text{I}\text{I}\text{C}={\text{R}}_{\text{C}\text{A}\text{L}}\times\frac{\text{min}\left(-{\text{M}\text{A}\text{E}}_{\text{c}\text{a}\text{l}},+{\text{M}\text{A}\text{E}}_{\text{c}\text{a}\text{l}}\right)}{\text{max}\left(-{\text{M}\text{A}\text{E}}_{\text{c}\text{a}\text{l}},+{\text{M}\text{A}\text{E}}_{\text{c}\text{a}\text{l}}\right)}$$

RCAL is the correlation coefficient between the experimental values and the calculated values of pIC_50_ in the calibration set. The negative and positive mean absolute errors are represented by -MAE and + MAE, respectively, and are assessed using the following equations:

8 $${}^{-}{\text{M}\text{A}\text{E}}_{\text{C}\text{A}\text{L}}=-\frac{1}{\text{N}}\sum_{y=1}^{{N}^{-}}\left|{\varDelta}_{\text{k}}\right|{\varDelta}_{\text{k}}<0,{}^{-}\text{N}\text{i}\text{s}\text{t}\text{h}\text{e}\text{n}\text{u}\text{m}\text{b}\text{e}\text{r}\text{o}\text{f}{\varDelta}_{\text{k}}<0$$

9 $${}^{+}{\text{M}\text{A}\text{E}}_{\text{C}\text{A}\text{L}}=+\frac{1}{\text{N}}\sum_{y=1}^{{N}^{+}}\left|{\varDelta}_{\text{k}}\right|{\varDelta}_{\text{k}}\ge0,{}^{+}\text{N}\text{i}\text{s}\text{t}\text{h}\text{e}\text{n}\text{u}\text{m}\text{b}\text{e}\text{r}\text{o}\text{f}{\varDelta}_{\text{k}}\ge0$$

10 $${\varDelta}_{\text{k}}={\text{O}\text{b}\text{s}}_{\text{k}}-{\text{C}\text{a}\text{l}\text{c}}_{\text{k}}$$

The index k ranges from 1 to N. Obs_k_ refers to the experimental values of pIC_50_, while Calc_k_ denotes the calculated values of pIC_50_.

### Applicability domain

According to OECD principles, a QSAR model must define an applicability domain (AD), which delineates the regions of physico-chemical, structural, or biological space where the QSAR model is expected to be useful and where predictions of endpoints are considered reliable^52^.

In CORAL, the AD is calculated based on the distribution of SMILES features in the training and calibration sets. The AD is represented as ‘DefectAK’, which is obtained using the following equations:

11 $${\text{D}\text{e}\text{f}\text{e}\text{c}\text{t}}_{{\text{A}}_{\text{K}}}=\frac{\left|{\text{P}}_{\text{T}\text{R}\text{N}}{(\text{A}}_{\text{K}})-{\text{P}}_{\text{C}\text{A}\text{L}}{(\text{A}}_{\text{K}})\right|}{{\text{N}}_{\text{T}\text{R}\text{N}}{(\text{A}}_{\text{K}})+{\text{N}}_{\text{C}\text{A}\text{L}}{(\text{A}}_{\text{K}})}\text{I}\text{f}{\text{A}}_{\text{K}}>0$$

$${\text{D}\text{e}\text{f}\text{e}\text{c}\text{t}}_{{\text{A}}_{\text{K}}}=1\text{I}\text{f}{\text{A}}_{\text{K}}=0$$

P_TRN_ and P_CAL_ represent the probability of a feature ‘A_K_’ in the training and calibration sets, respectively. Additionally, N_TRN_ and N_CAL_ indicate the number of occurrences of ‘A_K_’ in the training and calibration sets. To calculate the statistical defect (D), the following equations are applied:

12 $${\text{D}\text{e}\text{f}\text{e}\text{c}\text{t}}_{\text{M}\text{o}\text{l}\text{e}\text{c}\text{u}\text{l}\text{e}}=\sum_{\text{k}=1}^{N{\text{A}}_{}}{\text{D}\text{e}\text{f}\text{e}\text{c}\text{t}}_{{\text{A}}_{\text{K}}}$$

N_A_ represents the number of active SMILES features for the available compounds.

Finally, in CORAL, a compound is identified as an outlier if it satisfies inequality:

13 $${\text{D}\text{e}\text{f}\text{e}\text{c}\text{t}}_{\text{m}\text{o}\text{l}\text{e}\text{c}\text{u}\text{l}\text{e}}>2\times{\stackrel{-}{\text{D}\text{e}\text{f}\text{e}\text{c}\text{t}}}_{\text{T}\text{R}\text{N}}$$

${\stackrel{-}{\text{D}\text{e}\text{f}\text{e}\text{c}\text{t}}}_{\text{T}\text{R}\text{N}}$ represents the average statistical defect for the compounds in the training set.

### Model validation

One of the most crucial steps in constructing a model is validating it to assess its accuracy and reliability^53–55^. In this study, to verify the QSAR models and ensure their integrity and credibility, the following three methods were employed:

i. Internal Validation or Cross-Validation: This involves using the compounds in the training set to evaluate the models.
ii. External Validation: This method uses compounds in the validation set, which were not involved in model development, to test the models.
iii. Data Randomization or Y-Scrambling: This technique ensures that the modeling results are not due to chance.

The multiple statistical metrics used to validate the models include the correlation coefficient (R^2^), cross-validated correlation coefficient (Q^2^), concordance correlation coefficient (CCC), the index of ideality of correlation (IIC), ${Q}_{F1}^{2}$, ${Q}_{F2}^{2}$, and ${Q}_{F3}^{2}$, standard error of estimation (s), root mean square error (RMSE), mean absolute error (MAE), Fischer ratio (F), novel metrics (${r}_{m}^{2}$​), and Y-scrambling (${C}_{{R}_{p}^{2}}$). Additionally, several mathematical equations were employed to further validate the models, as shown in Table 2.

**Table 2:** The mathematical equations of statistical metrics as the criteria of the predictive potential.

Validation method	Criterion of the predictive potential
Internal	${R}^{2}=1-\frac{\sum{({Y}_{obs}-{Y}_{prd})}^{2}}{\sum{({Y}_{obs}-\stackrel{-}{Y})}^{2}}$
	$${Q}^{2}=1-\frac{\sum{({Y}_{prd}-{Y}_{obs})}^{2}}{\sum{({Y}_{obs}-{\overline{Y}}_{train})}^{2}}$$
External	${Q}_{F1}^{2}=1-\frac{\sum{({Y}_{per\left(test\right)}-{Y}_{obs\left(test\right)})}^{2}}{\sum{({Y}_{obs\left(test\right)}-{\overline{Y}}_{train})}^{2}}$
	$${Q}_{F2}^{2}=1-\frac{\sum{({Y}_{prd\left(test\right)}-{Y}_{obs\left(test\right)})}^{2}}{\sum{({Y}_{obs\left(test\right)}-{\overline{Y}}_{ext})}^{2}}$$
	$${Q}_{F3}^{2}=1-\frac{\sum{({Y}_{prd\left(test\right)}-{Y}_{obs\left(test\right)})}^{2}/{n}_{ext}}{\sum{({Y}_{obs\left(test\right)}-{\overline{Y}}_{train})}^{2}/{n}_{train}}$$
	$${r}_{m}^{2}={r}^{2}\times\left(1-\sqrt{{r}^{2}-{r}_{0}^{2}}\right)$$
	$$CCC=\frac{2\sum(X-\stackrel{-}{X}\left)\right(Y-\stackrel{-}{Y)}}{\sum{(X-\stackrel{-}{X})}^{2}+\sum{(Y-\stackrel{-}{Y})}^{2}+n({(\stackrel{-}{X}-\stackrel{-}{Y})}^{2}}$$
	$$MAE=\frac{1}{n}\times\sum\left|{Y}_{obs}-{Y}_{prd}\right|$$
Y-randomization	${cR}_{p}^{2}=R\sqrt{\left({R}^{2}-{R}_{r}^{2}\right)}$

### Molecular docking

Molecular docking is a structure-based technique that examines the binding interaction between two compounds. This method is widely used in drug discovery and development to assess how strongly two molecules bind within a biological system^56^. Docking analyzes the interactions between ligands and proteins, based on the Induced Fit Theory, to determine the best binding mode and the lowest binding free energy between the ligand and the protein, identifying the most favorable configuration^57^. The structures used in this process were drawn using ChemDraw 2D (Ultra 12), then optimized and energy-minimized using ChemDraw 3D.

The crystallographic structure of the required protein was obtained from two studies^39,58^. The two proteins acquired, with PDB codes 1SA0 and 3E22, have resolutions of 3.58Å and 3.80Å, respectively. Lower resolution indicates higher quality for molecular docking; therefore, protein 1SA0 was selected from the Protein Data Bank. This protein is tubulin, bound to colchicine as its active ligand. AutoDock Vina was used for the docking process, and the resulting data were visualized and analyzed using Discovery Studio Visualizer 2021.

## Results and discussion

### QSAR modeling

In the current research, the balance of correlation method was applied to construct QSAR models. A total of eight QSAR models were developed using two types of target functions: TF1 and TF2. To determine a suitable threshold value (T*) and the number of epochs (N*), a normal range for both was tested until optimal values were found. The values of T* and N* for splits 1, 3, and 4 were 1 and 10, respectively, while for split 2, the values were 1 and 15. These values remained consistent across the models for both TF1 and TF2.

Before model building commenced, seven compounds (49, 56, 74, 85, 112, 117, and 123) were identified as outliers due to an unacceptable range of standard deviation (> 3 S) and were therefore excluded from the dataset.

The following are the mathematical equations for the QSAR models predicting pIC_50_:

QSAR models without applying W_IIC_ (TF1):

14 $${\text{Split }}\;{\text{1}}:{\text{ pIC}}_{{{\text{5}}0}} = - 0.{{1992}}\left( { \pm \,0.0{\text{181}}} \right)\, + \,0.0{\text{718}}\left( { \pm \,0.000{\text{2}}} \right) \times {\text{DCW}}\left( {{\text{1}},{\text{1}}0} \right)$$

15 $${\text{Split}}\;{\text{ 2}}:{\text{ pIC}}_{{{\text{5}}0}} = - {{1}}.{{6562}}\left( { \pm \,0.0{\text{237}}} \right)\, + \,0.0{\text{816}}\left( { \pm \,0.000{\text{3}}} \right) \times {\text{DCW}}\left( {{\text{1}},{\text{15}}} \right)$$

16 $${\text{Split}}\;{{ 3}}:{\text{ pIC}}_{{{\text{5}}0}} \, = \,{{1}}.{\text{11}}00\left( { \pm \,0.00{\text{98}}} \right)\, + \,0.0{\text{746}}\left( { \pm \,0.000{\text{1}}} \right) \times {\text{DCW}}\left( {{\text{1}},{\text{1}}0} \right)$$

17 $${\text{Split }}\;{{4}}:{\text{ pIC}}_{{{\text{5}}0}} \, = \,0.0{\text{163}}\left( { \pm \,0.0{\text{111}}} \right)\, + \,0.0{\text{822}}\left( { \pm \,0.000{\text{1}}} \right) \times {\text{DCW}}\left( {{\text{1}},{\text{1}}0} \right)$$

QSAR models with applying W_IIC_ (TF2):

18 $${\text{Split }}\;{\text{1}}:{\text{ pIC}}_{{{\text{5}}0}} \, = \,{\text{2}}.{\text{2243}}\left( { \pm \,0.0{\text{143}}} \right)\, + \,0.0{\text{36}}0\left( { \pm \,0.000{\text{1}}} \right) \times {\text{DCW}}\left( {{\text{1}},{\text{1}}0} \right)$$

19 $${\text{Split}}\;{\text{ 2}}:{\text{ pIC}}_{{{\text{5}}0}} \, = \,{\text{2}}.{\text{34}}0{\text{8}}\left( { \pm \,0.0{\text{176}}} \right)\, + \,0.0{\text{311}}\left( { \pm \,0.000{\text{2}}} \right) \times {\text{DCW}}\left( {{\text{1}},{\text{15}}} \right)$$

20 $${\text{Split}}\;{\text{ 3}}:{\text{ pIC}}_{{{\text{5}}0}} \, = \,{\text{3}}.{\text{3343}}\left( { \pm \,0.0{\text{123}}} \right)\, + \,0.0{\text{187}}\left( { \pm \,0.000{\text{1}}} \right) \times {\text{DCW}}\left( {{\text{1}},{\text{1}}0} \right)$$

21 $${\text{Split }}\;{\text{4}}:{\text{ pIC}}_{{{\text{5}}0}} \, = \,{\text{2}}.{\text{4738}}\left( { \pm \,0.0{\text{187}}} \right)\, + \,0.0{\text{222}}\left( { \pm \,0.000{\text{1}}} \right) \times {\text{DCW}}\left( {{\text{1}},{\text{1}}0} \right)$$

In Table 3, the statistical outcomes of the QSAR models for eight splits are presented. The table shows that all designed QSAR models yielded desirable statistical results and met the requirements of various validation criteria. The QSAR model generated for split #2 emerged as the best-performing model (R^2^mValidation = 0.857; R^2^Validation = 0.872, Q^2^Validation = 0.8524). From the data in Table 3, it can be inferred that the models developed using IIC (TF2) produced more favorable and higher statistical results compared to those developed without IIC (TF1).

**Fig. 2:**
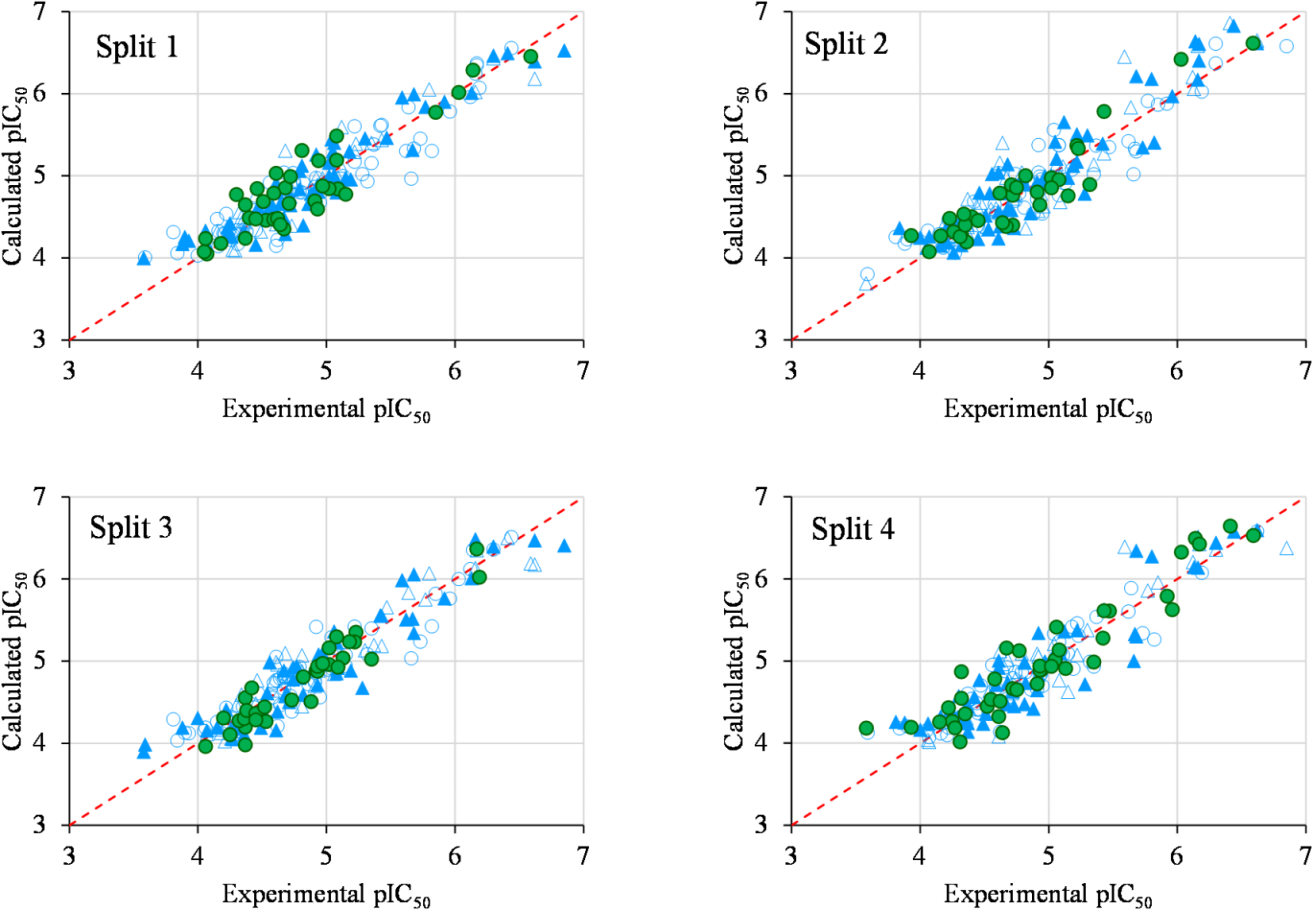
Graphical representation of the correlation between experimental and calculated pIC_50_ values of each split.

While the models constructed using TF2 showed lower statistical outcomes for the training sets, the calibration and validation results were significantly better. Consequently, it is evident that the models generated with IIC are statistically superior and more accurate for the current research and dataset. Additionally, Fig. 2 illustrates the correlation between the calculated pIC_50_ and experimental pIC_50_ for the four splits designed based on TF2.

**Table 3:** Statistical characteristics of each of the QSAR models for pIC50.

Split	Target function	Set	*n*	*R* ^2^	CCC	IIC	Q^2^	${\text{Q}}_{{\text{F}}_{1}}^{2}$	${\text{Q}}_{{\text{F}}_{2}}^{2}$	${\text{Q}}_{{\text{F}}_{3}}^{2}$	s	MAE	${r}_{m}^{2}$	CR^2^*p*
1	TF1	ATRN	59	0.9782	0.989	0.7793	0.9759				0.103	0.067		0.9729
		PTRN	53	0.9814	0.9793	0.7095	0.9789				0.149	0.122		0.9755
		CAL	39	0.5864	0.725	0.702	0.5172	0.2246	0.2207	0.4847	0.507	0.387	0.5653	0.5741
		VAL	35	0.5369	0.7254	0.6034	0.4634				0.4657	0.3781	0.4646	
	TF2	ATRN	59	0.8773	0.9347	0.8461	0.8697				0.245	0.19		0.8706
		PTRN	53	0.9007	0.9455	0.795	0.8917				0.228	0.188		0.8851
		CAL	39	0.8783	0.9361	0.9371	0.8585	0.8723	0.8716	0.9151	0.206	0.15	0.8218	0.8678
		VAL	35	0.8396	0.9142	0.5809	0.8199				0.2415	0.1957	0.7632	
2 Best	TF1	ATRN	56	0.9685	0.984	0.9162	0.9661				0.122	0.076		0.9548
		PTRN	57	0.9685	0.9622	0.8329	0.9656				0.205	0.154		0.9604
		CAL	41	0.6229	0.7577	0.6929	0.5666	0.3294	0.3279	0.3404	0.55	0.385	0.6007	0.6115
		VAL	32	0.637	0.7726	0.6469	0.5676				0.4316	0.3443	0.6296	
	TF2	ATRN	56	0.8715	0.9313	0.8692	0.862				0.246	0.194		0.8669
		PTRN	57	0.8713	0.9241	0.6669	0.8628				0.273	0.216		0.864
		CAL	41	0.8622	0.9186	0.9285	0.8457	0.8178	0.8174	0.8208	0.286	0.2	0.8507	0.8492
		VAL	32	0.872	0.9326	0.6767	0.8524				0.2122	0.1696	0.8570	
3	TF1	ATRN	59	0.9598	0.9795	0.947	0.9566				0.128	0.078		0.9528
		PTRN	54	0.9738	0.9721	0.8116	0.9701				0.182	0.136		0.9562
		CAL	41	0.7399	0.8529	0.7751	0.7094	0.6712	0.669	0.6352	0.408	0.314	0.7061	0.733
		VAL	32	0.7068	0.7751	0.6702	0.6469				0.4324	0.3252	0.5934	
	TF2	ATRN	59	0.8782	0.9351	0.7907	0.8708				0.222	0.172		0.8719
		PTRN	54	0.8882	0.9421	0.8716	0.8774				0.24	0.2		0.881
		CAL	41	0.9158	0.9554	0.9569	0.9058	0.9147	0.9141	0.9054	0.208	0.166	0.8344	0.9019
		VAL	32	0.913	0.9478	0.719	0.8995				0.1715	0.1339	0.8283	
4	TF1	ATRN	56	0.977	0.9884	0.7972	0.9749				0.087	0.05		0.9678
		PTRN	53	0.9639	0.9729	0.6659	0.9612				0.161	0.132		0.9477
		CAL	37	0.5878	0.7467	0.5089	0.5259	0.364	0.3446	0.2824	0.539	0.387	0.4371	0.5709
		VAL	40	0.681	0.7801	0.6628	0.6352				0.5692	0.4293	0.6107	
	TF2	ATRN	56	0.8546	0.9216	0.8607	0.844				0.218	0.165		0.8428
		PTRN	53	0.8624	0.9274	0.8498	0.8519				0.269	0.203		0.8581
		CAL	37	0.8685	0.9276	0.9319	0.8442	0.8511	0.8466	0.832	0.261	0.184	0.8455	0.8576
		VAL	40	0.8777	0.9347	0.5989	0.8653				0.2588	0.202	0.8381	

As discussed in Section “Applicability domain”, the AD of chalcone derivatives in the validation sets was predicted based on the defined defect criteria. Compounds were considered to fall within the domain of applicability if the defect in their SMILES representation was less than twice the mean defect of the training set ($2\times{\stackrel{-}{\text{D}\text{e}\text{f}\text{e}\text{c}\text{t}}}_{\text{T}\text{R}\text{N}}$). The values of ${\stackrel{-}{\text{D}\text{e}\text{f}\text{e}\text{c}\text{t}}}_{\text{T}\text{R}\text{N}}$ were found to be 0.99, 1.88, 1.69, and 1.57 for splits 1 to 4, respectively. A compound was classified as within the AD if its defect value was below 1.99, 3.76, 3.38, and 3.14 for splits 1 to 4, respectively. The percentages of compounds from the validation set that fell within the AD for each model split were 80%, 84%, 90%, and 85%, respectively. These results demonstrate that the developed models were able to predict the activity of over 80% of the new data points.

### Model interpretation

In CORAL, the correlation weights (CWs) of structural attributes (SAk) are calculated across three Monte Carlo runs, followed by a mechanistic interpretation based on the numerical statistics of these CWs. Descriptors that either increase or decrease the activity (pIC_50_) are identified consistently across all runs. If the CW(SAk) is positive in every Monte Carlo optimization run, the attribute is classified as an enhancing promoter, whereas a consistently negative CW(SAk) indicates a reducing promoter. Attributes with CWs that alternate between positive and negative across runs are considered inconsistent and are excluded as valuable descriptors^59^.

**Table 4:** Correlation weights (CWs) and identification of enhancing and reducing promoters for split 2.

No.	SAk	CWs Probe 1	CWs Probe 2	CWs Probe 3	NSs	NSc	NSv	Defect[SAk]	Comments
Promoter of endpoint increase									
Graph-based descriptors									
1	NNC-C…321.	0.191	0.433	0.394	56	57	41	0	The nearest neighbors’ codes for carbon equal to 321
2	PT2-C…3…	0.089	0.150	0.041	56	57	41	0	The number of paths of length 2 which started from carbon atom equal to 3
3	PT3-C…3…	0.151	0.159	0.403	56	56	40	0.0003	The number of paths of length 3 which started from carbon atom equal to 3
4	PT3-C…4…	0.237	0.437	0.189	56	57	41	0	The number of paths of length 3 which started from carbon atom equal to 4
5	PT3-O…3…	0.002	0.477	0.313	56	57	41	0	The number of paths of length 3 which started from oxygen atom equal to 3
SMILES-based descriptors									
	++++O—B2==	0.61485	2.64414	8.26606	56	57	41	0	Presence of oxygen with double bond
1	=…(…….	0.476	0.217	0.095	56	57	41	0	Presence of double bond with branching
2	=……….	0.041	0.045	0.180	56	57	41	0	Presence of double bond
3	C…(…….	0.296	0.083	0.336	56	57	41	0	Presence of aliphatic carbon with branching
4	C……….	0.072	0.002	0.075	56	57	41	0	Presence of aliphatic carbon
5	O…(…….	0.115	0.106	0.232	56	57	41	0	Presence of aliphatic oxygen with branching
6	O……….	0.442	0.150	0.275	56	57	41	0	Presence of aliphatic oxygen
7	O…=…(…	0.987	1.079	0.311	56	57	41	0	Presence of aliphatic oxygen with double bond and branching
Promoter of endpoint decrease									
Graph-based descriptors									
1	VS2-C…4…	-0.383	-0.919	-0.591	27	25	18	0.001	Valence shell of second order for carbon atom equal to 4
3	VS2-N…8…	-1.315	-0.244	-4.493	9	11	7	0.0006	Valence shell of second order for nitrogen atom equal to 8
2	NNC-N…330.	-2.241	-1.648	-0.967	11	11	9	0.0012	The nearest neighbors’ codes for nitrogen equal to 330
4	C5…AH.2…	-1.112	-1.234	-0.143	19	19	14	0.0001	Presence of five-member ring with two heteroatoms
SMILES-based descriptors									
1	c…O…….	-0.083	-1.209	-1.337	49	52	37	0.0003	Presence of aromatic carbon with aliphatic oxygen atom
2	c…3…….	-0.161	-0.543	-1.011	30	33	22	0	Presence of aromatic carbon in ring number 3
3	c…1…(…	-0.303	-0.827	-1.327	18	15	14	0.0006	Presence of aromatic carbon in ring number 1 with branching
4	C…N…(…	-1.129	-1.486	-0.744	12	8	10	0.0013	Presence of aliphatic carbon with aliphatic nitrogen with branching
5	c…3…c…	-0.736	-0.540	-0.070	12	13	11	0.0023	Presence of aromatic carbon in ring number 3 connected to the other aromatic carbon
6	n…2…….	-0.077	-0.033	-0.078	11	13	8	0.0001	Presence of aromatic nitrogen in ring number 2
7	s…(…….	-2.368	-1.512	-0.724	10	5	8	0.0009	Presence of aromatic sulfur with branching
8	\…c…4…	-2.512	-0.368	-0.561	9	6	7	0.0006	Presence of aromatic carbon in ring number 4
9	c…3…(…	-0.462	-0.156	-0.880	9	8	9	0.0033	Presence of aromatic carbon in ring number 3 with branching
10	c…5…….	-0.649	-0.360	-0.317	9	11	5	0.0028	Presence of aromatic carbon in ring number 5

In Table 4, the attributes and their corresponding CWs for Split #2, representing the leading model, are presented. The graph-based descriptors associated with increased pIC_50_ include the Nearest Neighbor Codes for Carbon (NNC-C) equal to 321 (NNC-C…321). This descriptor highlights the importance of a carbon atom in the molecular structure with three connections: two bonds to other carbon atoms and one bond to a non-carbon atom. Specifically, this descriptor points to the presence of a carbonyl group within the chalcone scaffold, which serves as a key functional group contributing to the biological activity of the compounds.

The number of paths of length 2 starting from a carbon atom equal to 3 (PT2-C…3…), the number of paths of length 3 starting from a carbon atom equal to 3 (PT3-C…3…), the number of paths of length 3 starting from a carbon atom equal to 4 (PT3-C…4…), and the number of paths of length 3 starting from an oxygen atom equal to 3 (PT3-O…3…) are additional descriptors associated with increased pIC_50_. These structural attributes highlight specific connectivity patterns in the molecular framework and were identified as the most effective factors contributing to the enhanced inhibitory activity of chalcone derivatives against HT-29 colon cancer cells.

The SMILES-based descriptors are more interpretable. These descriptors include the presence of oxygen and a double bond (+++O—B2==), a double bond with branching (=…(…….), a double bond (=……….), an aliphatic carbon with branching (C…(…….), an aliphatic carbon (C……….), an aliphatic oxygen with branching (O…(…….), an aliphatic oxygen (O……….), and an aliphatic oxygen with a double bond and branching (O…=…(….). All these descriptors further highlight the presence of the carbonyl group within the chalcone structure, which is a key feature of the scaffold. Additionally, the double bond is indicative of the vinyl group in the chalcone structure, emphasizing its importance in the biological activity.

The graph-based descriptors associated with decreased pIC_50_ include: the valence shell of second order for carbon atoms equal to 4 (VS2-C…4…); the valence shell of second order for nitrogen atoms equal to 8 (VS2-N…8…). Moreover, the nearest neighbor codes for nitrogen equal to 330 (NNC-N…330…) specifically points to the presence of nitrogen with three carbon connections that lack a non-carbon connection (e.g., N(CH3)_2_​), which can be observed in compounds 42 and 43 (Table S1), which exhibit the lowest pIC_50_ values in the dataset. The pIC_50_​ values for these compounds are 3.59 and 3.58, respectively.

The graph-based descriptors associated with decreased pIC_50_​ include: the valence shell of second order for carbon atoms equal to 4 (VS2-C…4…); the valence shell of second order for nitrogen atoms equal to 8 (VS2-N…8…); and a five-member ring containing two heteroatoms (C5…AH.2…). Moreover, the nearest neighbor codes for nitrogen equal to 330 (NNC-N…330…) specifically point to the presence of nitrogen with three carbon connections that lack a non-carbon connection (e.g., N(CH3)_2_​), which can be observed in compounds 42 and 43 (Table S1). These compounds exhibit the lowest pIC_50_ values in the dataset, with pIC_50_ ​ values of 3.59 and 3.58, respectively.

The SMILES-based descriptors associated with decreased pIC_50_ include: the presence of an aromatic carbon with an aliphatic oxygen atom (c…O…….); an aromatic carbon in a ring numbered 3 (c…3…….); an aromatic carbon in a ring numbered 1 with branching (c…1…(…)); an aliphatic carbon with an aliphatic nitrogen and branching (C…N…(…)); an aromatic carbon in ring number 3 connected to another aromatic carbon (c…3…c…); an aromatic nitrogen in a ring numbered 2 (n…2…….); an aromatic sulfur with branching (s…(…….)); an aromatic carbon in a ring numbered 4 (c…4…); an aromatic carbon in ring number 3 with branching (c…3…(…)); and an aromatic carbon in ring number 5 (c…5…….).

Several attributes demonstrated that the core chalcone scaffold is crucial for potential inhibitory activity, as indicated by the presence of oxygen and a double bond (++++O—B2==), a double bond with branching (=…(…….), a double bond (=……….), an aliphatic carbon with branching (C…(…….), an aliphatic carbon (C……….), an aliphatic oxygen with branching (O…(…….), an aliphatic oxygen (O……….), and an aliphatic oxygen with a double bond and branching (O…=…(…). These features suggest the importance of the carbonyl functionality within the chalcone scaffold.

### Proposed chalcone derivatives via in Silico screening

A set of compounds, particularly synthesized chalcone derivatives with unknown pIC50 values against HT-29 human colon adenocarcinoma cell lines, was identified through in silico screening of the ChEMBL dataset, based on their potential to modulate endpoint promoters^49,50^. All of these compounds are within the applicability domain of the proposed model.

The pIC_50_ values for these 11 compounds were predicted using Split #2 as the best model. Table 5 presents the molecular structures, ChEMBL IDs, and the predicted pIC_50_ values for each molecule. These compounds were subsequently subjected to molecular docking studies to evaluate their potential interactions and biological relevance.

**Table 5:** ChEMBL ids, molecular structures, and predicted pIC_50_ values of compounds extracted from the chembl database.

No.	ChEMBL ID	Structure	Prd. pIC_50_
1	CHEMBL552766		6.28
2	CHEMBL3924305		8.44
3	CHEMBL3943284		9.32
4	CHEMBL554966		6.46
5	CHEMBL3934317		6.23
6	CHEMBL3896972		6.60
7	CHEMBL3906339		6.24
8	CHEMBL538187		6.74
9	CHEMBL539438		6.46
10	CHEMBL3916924		6.56
11	CHEMBL552539		6.55

### Molecular Docking investigation

Based on the previous data, the eleven compounds identified from enhancing/reducing promoters were evaluated for their potential inhibitory effects. To ensure a valid comparison, a known reference compound, colchicine (an active ligand of tubulin) was selected for co-crystallization and redocking with tubulin. This step serves as an authenticity check to validate the docking process. The docking simulations were performed using AutoDock Vina.

**Fig. 3:**
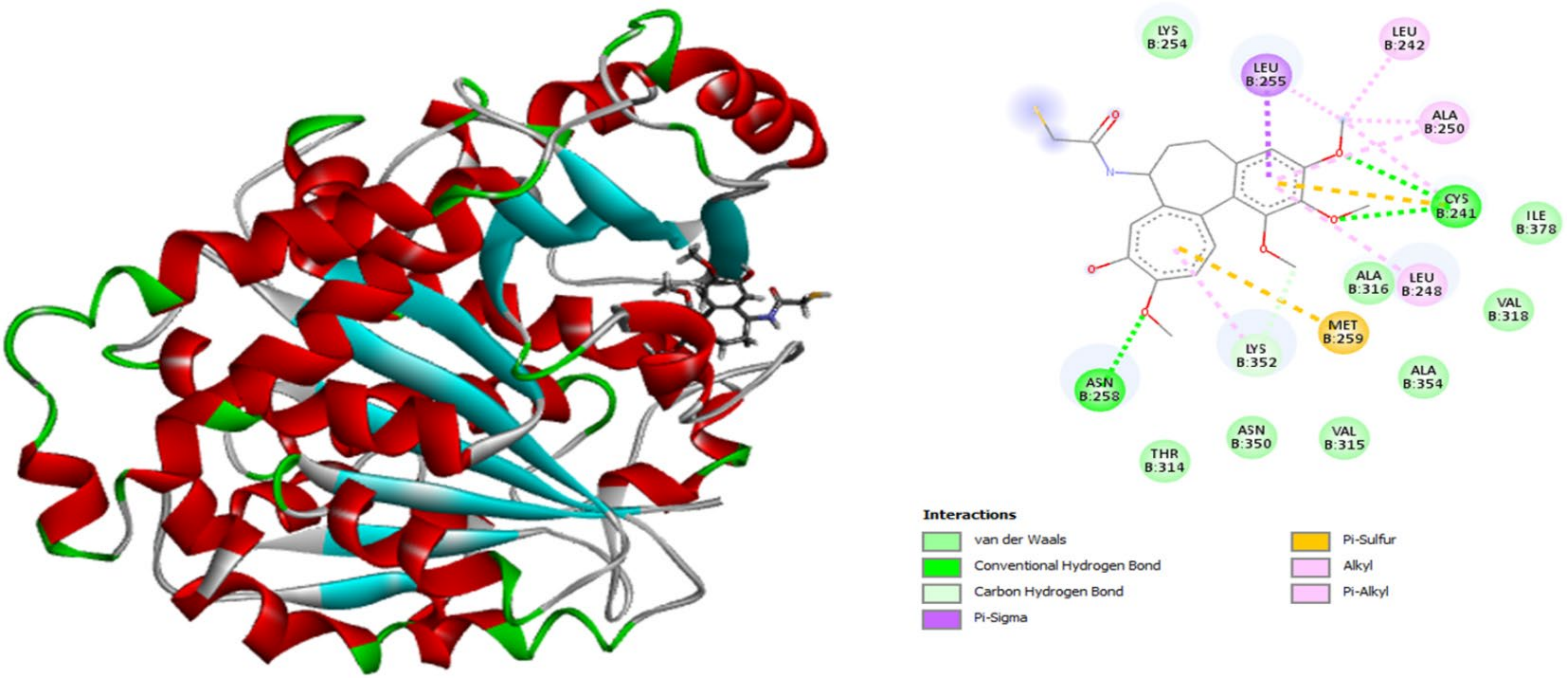
Superimposition of colchicine and tubulin, with 2D schematic of interactions between the two compounds.

According to the results from the docking process, the active site of tubulin (Fig. 3) includes amino acids such as ASN B:258, MET B:259, CYS B:241, LYS B:352, LEU B:255, LEU B:248, ALA B:250, and LEU B:242. These residues form strong bonds with the ligand through key interactions, including hydrogen bonds, van der Waals forces, pi-sigma, pi-sulfur, and alkyl interactions. A detailed analysis of the ligand-protein interactions reveals that the enhancing promoters, which increase pIC_50_ values, are crucially involved in these significant interactions.

The numerical values of binding energy with tubulin (pdb:1SA0) for these compounds, along with the active ligand, are presented in Table 6. The compound with the best binding energy is compound 1, with a value of -9.1 kcal/mol. The docking results indicate that lower binding energy corresponds to higher binding affinity between the ligand and receptor. All of these compounds demonstrated acceptable binding energies compared to the active ligand.

**Fig. 4:**
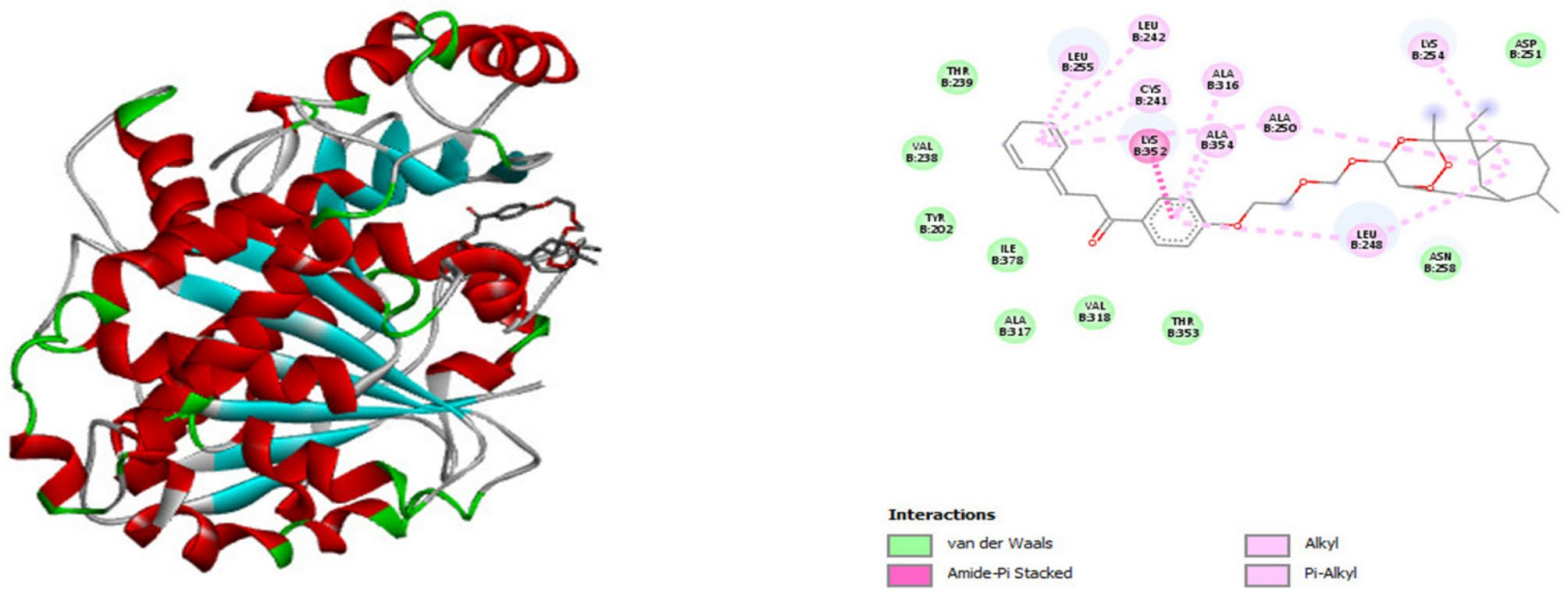
Superimposition of Compound No. 1 and tubulin, with 2D Schematic of interactions between the two compounds.

Figure 3 illustrates the superimposition of the active ligand with the active site of tubulin (1SA0) and shows the 2D interactions between the ligand and the receptor’s amino acids. Figure 4 provides both a 3D representation and 2D interactions of compound number 1, which exhibits the best binding energy, as detailed in Table 6.

However, the study presents only the interactions of the active ligand and Ligand No.1; interactions of other compounds, both in 2D and 3D, have been observed and discussed. The results regarding the amino acid residues and their interactions are summarized in Table 6.

Based on the docking results presented in Table 6 and the structural data in Table 5, it can be inferred that the active site of the target protein may not be sufficiently large to accommodate ligands with two polycyclic ends (e.g., Ligands 2 and 3, which show the weakest binding affinities). This indicates that the protein’s amino acid residues must provide adequate space to form strong interactions with the ligands. Moreover, specific interactions such as amide-π stacking, conventional hydrogen bonds, carbon-hydrogen bonds, π-sigma interactions, and π-sulfur interactions proved to be critical during the docking process. Ligands with high binding affinity typically display at least one of these interactions. Notably, certain amino acid residues, including LYS B:254, LYS B:352, CYS B:352, LEU B:248, LEU B:255, and ALA B:250, were involved in nearly all interactions, highlighting their importance.

**Table 6:** Binding energy values for the Docking interactions of the extracted compounds with tubulin.

No.	Binding affinity(kcal/mol)	Amino acid residue	Type of interactions
AL (Colchicine)	-7.7	ASN B:258, CYS B:241, MET B:259, LEU B:255, LEU B:242, ALA B: 250, LEU B:248, LYS B:352	Conventional Hydrogen Bond, Pi-Sigma, Pi-Sulfur, Carbon Hydrogen Bond, Alkyl and Van der Waals
1	-9.1	LYS B:352, LEU B:255, LEU B:242, LEU B:248, CYS B:241, ALA B:250, ALA B:354, LYS B:254	Amide-Pi Stacked, Alkyl, Pi-Alkyl and Van der Waals
2	-5.2	LYS B:352, LEU B:255, LEU B:248, ASN B:258, ALA B:250, ALA B:316, ASP B:251, LYS B:254	Conventional Hydrogen Bond, Pi-Cation, Pi-Sigma, Pi-Alkyl and Van der Waals
3	-5.0	LYS B:352, ASN B: 258, VAL B:315, ALA B:316, LEU B:255, CYS B:241, ASP B:251, LEU B:242	Conventional Hydrogen Bond, Carbon Hydrogen Bond, Alkyl and Van der Waals
4	-8.6	CYS B:241, LEU B:255, LYS B:352, ALA B:250, LEU B:242, ALA B:354, LEU B:248, ASP B:251	Pi-Sulfur, Pi-Sigma, Amide-Pi Stacked, Alkyl, Pi-Alkyl and Van der Waals
5	-6.9	ALA B:250, LYS B:254, CYS B:241, LEU B:248, LEU B:255, VAL B:318, ILE B:378, LYS B:352	Conventional Hydrogen Bond, Pi-Sigma, Pi-Sulfur, Pi-Cation, Carbon Hydrogen Bond, Alkyl, Pi-Alkyl and Van der Waals
6	-7.8	LYS B:254, LEU B:255, ALA B:316, VAL B:318, LEU B:248, ILE B:378, CYS B:241, ALA B:250	Pi-Cation, Pi-Sigma, Alkyl, Pi-Alkyl and Van der Waals
7	-7.5	CYS B:241, LEU B:255, ASN B:258, LYS B:352, ALA B:250, VAL B:238, LEU B:242, VAL B:318	Pi-Sigma, Pi-Sulfur, Carbon Hydrogen Bond, Alkyl, Pi-Alkyl and Van der Waals
8	-7.5	LYS B:352, CYS B:241, LYS B:254, LEU B:248, ALA B:316, ILE B:378, LEU B:255, ALA B:354	Amide-Pi Stacked, Pi-Sulfur, Alkyl, Pi-Alkyl and Van der Waals
9	-8.4	ALA B:316, LEU B:255, ASN B:258, VAL B:238, PHE B:169, TYR B:202, CYS B:241, LYS B:352	Pi-Sigma, Carbon Hydrogen Bond, Alkyl, Pi-Alkyl and Van der Waals
10	-6.8	LYS B:352, ALA B:316, ALA B:250, LEU B:248, ASN B:249, LEU B:255, LYS B:254, THR B:353	Carbon Hydrogen Bond, Alkyl and Van der Waals
11	-8.2	LYS B:352, CYS B:241, ALA B:250, LEU B:255, ALA B:316, ASN B:258, ASP B:251, LYS B:254	Conventional Hydrogen Bond, Carbon Hydrogen Bond, Alkyl and Van der Waals

Comparing the pIC_50_ values of the proposed compounds with their binding affinities to tubulin (PDB: 1SA0) reveals a lack of significant correlation. Although docking studies offer valuable insights into potential binding interactions, the correlation between docking binding energy and pIC_50_ values may be limited by factors such as the complexity of biological systems, model limitations, and experimental variability. These considerations are important when interpreting docking results and their relationship to biological activity.

### Docking protocol validation

**Fig. 5:**
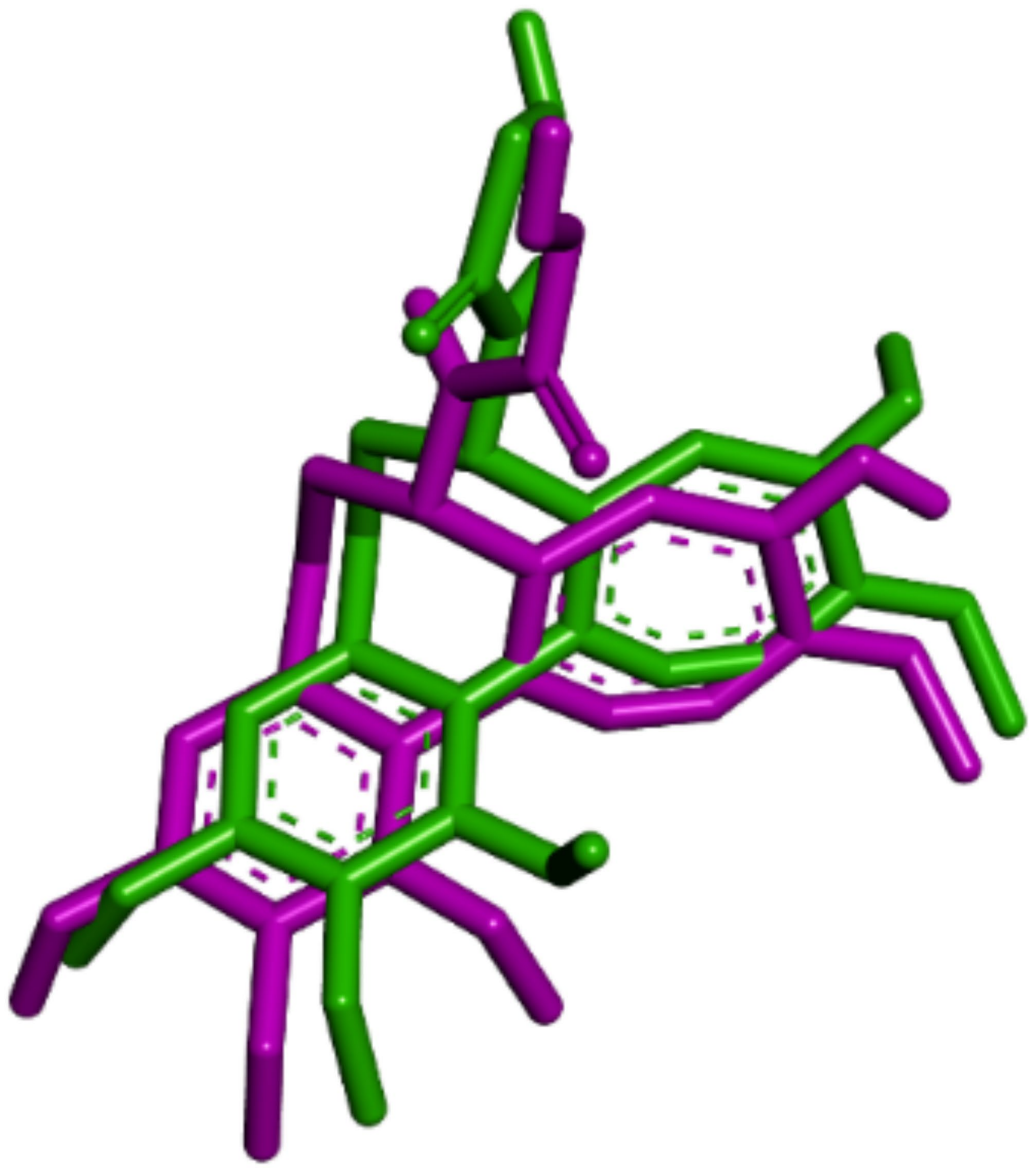
Docking validation, a comparison of the redocked binding mode (magenta) and the co-crystallized pose (forest green) of colchicine.

The accuracy of the docking protocol was confirmed through the re-docking of the native ligand (Colchicine) into the pr*ot*ein receptor’s binding site ((PDB: 1SA0). An RMSD value of 1.324 Å was identified as the standard threshold, indicating the protocol’s stability and reliability (Fig. 5).

### Comparison with previous QSAR studies

There is one report on QSAR modeling of chalcone derivatives as HT-29 inhibitors. According to a review of the literature, Rybka et al. developed a QSAR model in 2014 to predict the inhibitory activity of chalcone derivatives against HT-29^37^. While this is the closest research to our study, no previous work has constructed QSAR models using SMILES notations to evaluate the inhibitory effects of chalcone derivatives on HT-29. Other QSAR studies have been conducted using various methods to assess the inhibitory activity of chalcone derivatives on different cell lines^60,61^. Rybka et al. gathered 162 chalcone molecules from various sources and developed a QSAR model using a training set of 136 compounds and a test set of 19 compounds. The IC50 values were obtained through the MTT assay, and outliers were excluded based on excessively high prediction errors. Their final QSAR model, built using the Enhanced Replacement Method (ERM), incorporated seven molecular descriptors chosen from over 1,400 potential descriptors, including constitutional, topological, geometrical, and 3D-MoRSE descriptors. The most significant descriptors were the number of 10-membered rings (nR10) and a BCUT descriptor weighted by van der Waals volume (BELm1). The statistical performance of the model was as follows: R2train = 0.896; Q2 = 0.885; R2 test = 0.856; Strain = 0.25 and Stest = 0.22.

In comparison, we constructed a QSAR model using 193 chalcone derivatives. Our model yielded R^2^_train_ = 0.871; R^2^_test_ = 0.872, and S_train_=0.24 and S_test_=0.21.

The dataset used for our model is larger than the one reported by Rybka et al., and the statistical parameters of our model are comparable to theirs. These differences, particularly in dataset size and performance metrics, contribute to the enhanced accuracy and predictability of our QSAR model compared to previous studies. Unlike previous studies, the models generated using CORAL do not require molecular optimization, which simplifies the modeling process. Furthermore, CORAL uses simpler increasing and decreasing molecular descriptors, making the QSAR models both efficient and easy to interpret. This contrasts with the more complex descriptors employed in other studies, such as those based on geometrical or quantum-mechanical properties, which often require pre-optimization of molecular structures. These differences contribute to the efficiency and accuracy of our models while maintaining robustness in predicting the inhibitory activity of chalcone derivatives.

## Conclusion

This study presents a robust QSAR model developed to predict the inhibitory activity of 193 chalcone derivatives against HT-29 adenocarcinoma cell lines using the Monte Carlo method with CORAL software. Two target function for monte Carlo optimization were applied: TF1 (without WIIC) and TF2 (with WIIC = 0.2). The inclusion of the index of ideality of correlation (IIC) significantly improved the accuracy and reliability of the models, as the TF2 models consistently outperformed those developed without WIIC. The models’ predictive performance was evaluated using several key statistical metrics, such as R^2^, Q^2^, and IIC, alongside error estimates like MAE and RMSE. Additionally, the applicability domain (AD) was assessed to identify and exclude outliers, ensuring robust predictions. Structural features influencing inhibitory activity were also identified and used to guide the prediction of pIC50 for other chalcone derivatives. Finally, molecular docking studies targeting the active site of tubulin (PDB ID: 1SA0) provided mechanistic validation, supporting the relevance of the QSAR model in explaining the interaction.

## Electronic supplementary material

Below is the link to the electronic supplementary material.

Supplementary Material 1

Supplementary Material 2

## Data Availability

Data is provided within the manuscript or supplementary information files.
