# Supplementary material for: SMILES-based QSAR and molecular docking studies of chalcone analogues as potential anti-colon cancer
Source: Sci Rep. 2025 Feb 24;15:6573. doi: 10.1038/s41598-025-91338-9 (PMC11850874; doi:10.1038/s41598-025-91338-9)
Supplement: Supplementary file 1 — Supplementary Material 1 [file 41598_2025_91338_MOESM1_ESM.docx]

**Table S2.** SMILES notation, distribution of compounds in splits, DCWs, experimental and calculated pIC_50_ of chalcones (+, -, # and * indicate the training, the invisible training, the calibration and the validation sets respectively.)

| ID | SMILES | pIC50 (Exp.) | Splits | | | | DCW | | | | pIC50(Calc.) | | | |
| --- | --- | --- | --- | --- | --- | --- | --- | --- | --- | --- | --- | --- | --- | --- |
|  |  |  | 1 | 2 | 3 | 4 | 1 | 2 | 3 | 4 | 1 | 2 | 3 | 4 |
| 1 | Nc1ccc(cc1)C(=O)\C=C\c2ccc(Cl)cc2Cl | 4.4 | * | * | + | + | 66.00 | 68.85 | 76.14 | 54.22 | 4.49 | 4.50 | 4.35 | 4.54 |
| 2 | COc1ccc(cc1)C(=O)\C=C\c2ccc(Cl)cc2Cl | 4.72 | - | * | - | * | 70.94 | 76.38 | 81.60 | 58.47 | 4.64 | 4.76 | 4.54 | 4.67 |
| 3 | Oc1ccccc1C(=O)\C=C\c2ccc(Cl)cc2Cl | 4.37 | * | # | * | + | 71.13 | 74.99 | 82.00 | 56.36 | 4.65 | 4.71 | 4.55 | 4.60 |
| 4 | Nc1ccc(cc1)C(=O)\C=C\c2ccccc2Cl | 4.53 | * | - | * | + | 65.00 | 64.54 | 73.93 | 48.08 | 4.46 | 4.35 | 4.27 | 4.36 |
| 5 | COc1ccc(\C=C\C(=O)c2ccccc2)cc1 | 4.62 | + | - | + | + | 57.32 | 65.54 | 72.33 | 40.39 | 4.22 | 4.38 | 4.21 | 4.14 |
| 6 | COc1ccc(\C=C\C(=O)c2ccc(N)cc2)cc1 | 4.25 | - | + | * | * | 63.86 | 61.79 | 69.43 | 44.81 | 4.42 | 4.25 | 4.11 | 4.27 |
| 7 | COc1ccc(\C=C\C(=O)c2cccc(N)c2)cc1 | 4.37 | # | # | * | - | 64.30 | 63.06 | 72.02 | 44.14 | 4.43 | 4.30 | 4.20 | 4.25 |
| 8 | COc1ccc(\C=C\C(=O)c2ccc(OC)cc2)cc1 | 4.31 | + | # | # | - | 61.18 | 66.69 | 73.45 | 44.95 | 4.34 | 4.42 | 4.25 | 4.27 |
| 9 | COc1ccc(\C=C\C(=O)c2ccccc2O)cc1 | 4.23 | - | * | + | + | 62.35 | 68.28 | 73.88 | 45.99 | 4.37 | 4.48 | 4.27 | 4.30 |
| 10 | COc1ccc(\C=C\C(=O)c2cc(F)cc(F)c2)cc1 | 4 | + | - | - | - | 51.26 | 61.60 | 75.12 | 41.28 | 4.03 | 4.24 | 4.31 | 4.16 |
| 11 | Nc1ccc(cc1)C(=O)\C=C\c2ccccc2F | 4.32 | # | * | * | * | 68.25 | 62.72 | 74.22 | 54.48 | 4.56 | 4.28 | 4.28 | 4.55 |
| 12 | COc1ccc(cc1)C(=O)\C=C\c2ccccc2F | 4.72 | - | * | + | - | 74.29 | 65.98 | 82.68 | 57.25 | 4.74 | 4.40 | 4.58 | 4.63 |
| 13 | Oc1ccc(\C=C\C(=O)c2ccccc2)cc1 | 4.61 | + | - | - | # | 55.02 | 61.36 | 70.87 | 38.54 | 4.15 | 4.24 | 4.16 | 4.08 |
| 14 | COc1ccc(cc1)C(=O)\C=C\c2ccc(O)cc2 | 4.73 | # | - | + | - | 62.99 | 64.96 | 77.35 | 51.16 | 4.39 | 4.36 | 4.39 | 4.45 |
| 15 | COc1ccc(\C=C\C(=O)c2ccccc2)c(OC)c1 | 3.88 | - | + | - | - | 55.75 | 59.55 | 71.76 | 44.18 | 4.17 | 4.17 | 4.19 | 4.25 |
| 16 | COc1cccc(\C=C\C(=O)c2ccc(F)c(F)c2)c1 | 4.27 | # | # | - | - | 53.41 | 62.36 | 67.86 | 46.41 | 4.10 | 4.27 | 4.05 | 4.31 |
| 17 | COc1cccc(\C=C\C(=O)c2ccc(N)cc2)c1OC | 4.16 | + | - | # | - | 60.88 | 58.85 | 72.31 | 46.65 | 4.33 | 4.15 | 4.21 | 4.32 |
| 18 | COc1cccc(\C=C\C(=O)c2cccc(N)c2)c1OC | 4.36 | # | * | * | # | 61.32 | 60.11 | 74.90 | 45.98 | 4.34 | 4.19 | 4.30 | 4.30 |
| 19 | COc1ccc(cc1)C(=O)\C=C\c2cccc(OC)c2OC | 4.38 | + | + | * | # | 60.60 | 67.04 | 77.68 | 49.92 | 4.32 | 4.44 | 4.40 | 4.42 |
| 20 | FC(F)(F)c1ccccc1\C=C\C(=O)c2ccccc2 | 4.37 | + | # | * | - | 58.14 | 66.05 | 65.93 | 40.42 | 4.24 | 4.40 | 3.98 | 4.14 |
| 21 | COc1ccc(cc1)C(=O)\C=C\c2ccccc2C(F)(F)F | 4.44 | # | # | + | + | 74.14 | 72.56 | 78.73 | 52.41 | 4.74 | 4.63 | 4.44 | 4.49 |
| 22 | Nc1ccc(cc1)C(=O)\C=C\c2ccccc2Br | 4.45 | + | - | # | + | 61.30 | 61.17 | 80.12 | 49.99 | 4.34 | 4.23 | 4.49 | 4.42 |
| 23 | COc1ccc(cc1)C(=O)\C=C\c2ccccc2Br | 4.59 | * | + | + | + | 65.44 | 70.10 | 87.05 | 58.58 | 4.47 | 4.54 | 4.73 | 4.67 |
| 24 | COc1ccc(cc1)C(=O)\C=C\c2ccc(F)cc2F | 4.95 | + | + | + | + | 72.04 | 70.02 | 82.23 | 59.53 | 4.67 | 4.54 | 4.56 | 4.70 |
| 25 | COc1ccc(cc1OC)C(=O)\C=C\c2ccc(Cl)cc2Cl | 4.73 | # | - | * | + | 77.04 | 76.40 | 81.30 | 58.06 | 4.83 | 4.76 | 4.53 | 4.65 |
| 26 | COc1ccc(cc1OC)C(=O)\C=C\c2ccc(cc2)N(C)C | 4.3 | * | - | # | - | 75.18 | 66.69 | 79.48 | 55.29 | 4.77 | 4.42 | 4.46 | 4.57 |
| 27 | COc1ccc(cc1OC)C(=O)\C=C\c2ccccc2Cl | 4.81 | + | + | # | - | 76.04 | 72.10 | 79.09 | 51.92 | 4.80 | 4.61 | 4.45 | 4.47 |
| 28 | COc1ccc(\C=C\C(=O)c2ccc(OC)c(OC)c2)cc1 | 4.26 | - | * | - | # | 58.62 | 63.66 | 71.22 | 45.32 | 4.26 | 4.32 | 4.17 | 4.28 |
| 29 | COc1ccc(\C=C\C(=O)c2ccc(OC)c(OC)c2)c(OC)c1 | 4.18 | * | + | # | # | 55.99 | 58.17 | 69.92 | 47.90 | 4.18 | 4.12 | 4.12 | 4.36 |
| 30 | COc1ccc(cc1OC)C(=O)\C=C\c2cccc(OC)c2OC | 4.45 | * | * | * | + | 65.64 | 67.56 | 76.65 | 48.31 | 4.48 | 4.45 | 4.36 | 4.37 |
| 31 | COc1ccc(cc1OC)C(=O)\C=C\c2ccccc2Br | 4.86 | - | - | + | + | 71.54 | 70.13 | 86.75 | 58.17 | 4.66 | 4.54 | 4.72 | 4.66 |
| 32 | O=C(\C=C\c1ccccc1)c2ccccc2 | 4.31 | + | * | + | * | 55.81 | 61.95 | 70.63 | 36.27 | 4.17 | 4.26 | 4.15 | 4.02 |
| 33 | Cc1ccc(cc1)C(=O)\C=C\c2ccccc2 | 4.07 | - | * | - | + | 55.47 | 56.77 | 70.76 | 38.15 | 4.16 | 4.07 | 4.15 | 4.07 |
| 34 | COc1ccc(cc1)C(=O)\C=C\c2ccccc2 | 3.81 | + | + | + | - | 60.40 | 61.87 | 74.67 | 44.54 | 4.31 | 4.25 | 4.29 | 4.26 |
| 35 | Clc1ccccc1\C=C\C(=O)c2ccccc2 | 4.26 | # | + | - | + | 58.88 | 61.35 | 68.05 | 43.67 | 4.27 | 4.24 | 4.06 | 4.23 |
| 36 | Oc1ccc(cc1)C(=O)\C=C\c2ccccc2Cl | 4.22 | + | + | - | * | 67.64 | 67.89 | 77.93 | 50.47 | 4.54 | 4.47 | 4.41 | 4.43 |
| 37 | Cc1cccc(\C=C\C(=O)c2ccccc2)c1 | 4.07 | * | - | # | # | 51.82 | 62.00 | 69.58 | 36.25 | 4.05 | 4.26 | 4.11 | 4.02 |
| 38 | Cc1cccc(\C=C\C(=O)c2ccc(O)cc2)c1 | 4.29 | # | + | - | - | 53.23 | 59.22 | 71.50 | 46.58 | 4.09 | 4.16 | 4.18 | 4.32 |
| 39 | Cc1ccc(cc1)C(=O)\C=C\c2cccc(C)c2 | 4.16 | + | * | # | + | 54.94 | 62.29 | 71.17 | 39.71 | 4.14 | 4.27 | 4.17 | 4.12 |
| 40 | COc1ccc(cc1)C(=O)\C=C\c2cccc(C)c2 | 4.2 | + | # | * | - | 59.87 | 67.38 | 75.08 | 46.09 | 4.30 | 4.45 | 4.31 | 4.30 |
| 41 | COc1ccc(\C=C\C(=O)c2ccc(C)cc2)cc1 | 3.93 | - | * | + | * | 57.02 | 62.38 | 69.82 | 42.31 | 4.21 | 4.27 | 4.12 | 4.19 |
| 42 | CN(C)c1ccc(\C=C\C(=O)c2ccccc2)cc1 | 3.59 | + | + | - | + | 50.51 | 48.93 | 66.01 | 40.07 | 4.01 | 3.80 | 3.99 | 4.13 |
| 43 | CN(C)c1ccc(\C=C\C(=O)c2ccc(C)cc2)cc1 | 3.58 | - | # | - | * | 50.20 | 45.78 | 63.50 | 41.98 | 4.00 | 3.69 | 3.90 | 4.18 |
| 44 | Oc1ccccc1\C=C\C(=O)c2ccccc2 | 4.05 | * | - | + | + | 52.72 | 58.21 | 70.68 | 41.12 | 4.08 | 4.13 | 4.15 | 4.16 |
| 45 | Cc1ccc(cc1)C(=O)\C=C\c2ccccc2O | 4.26 | + | + | + | - | 58.65 | 62.07 | 75.53 | 44.57 | 4.26 | 4.26 | 4.32 | 4.26 |
| 46 | COc1ccc(cc1)C(=O)\C=C\c2ccccc2O | 4.35 | # | * | + | * | 64.83 | 66.00 | 78.90 | 47.93 | 4.45 | 4.40 | 4.44 | 4.36 |
| 47 | Clc1cccc(\C=C\C(=O)c2ccccc2)c1 | 4.49 | # | + | - | - | 60.59 | 65.44 | 71.70 | 43.81 | 4.32 | 4.38 | 4.19 | 4.24 |
| 48 | CCOc1ccc(O)c(c1)C(=O)\C=C\c2ccc(C)s2 | 4.36 | - | - | - | - | 60.36 | 61.10 | 69.48 | 43.96 | 4.31 | 4.23 | 4.11 | 4.24 |
| 50 | CCCOc1ccc(O)c(c1)C(=O)\C=C\c2ccc(C)s2 | 3.9 | - | + | + | + | 58.53 | 61.07 | 70.06 | 42.66 | 4.26 | 4.23 | 4.13 | 4.20 |
| 51 | CC(C)Oc1ccc(O)c(c1)C(=O)\C=C\c2ccc(C)s2 | 4.52 | + | # | * | * | 68.63 | 65.47 | 78.89 | 51.07 | 4.57 | 4.38 | 4.44 | 4.45 |
| 52 | CCCCOc1ccc(O)c(c1)C(=O)\C=C\c2ccc(C)s2 | 4.15 | # | + | - | * | 60.33 | 62.86 | 71.79 | 44.60 | 4.31 | 4.29 | 4.19 | 4.26 |
| 53 | CC(C)COc1ccc(O)c(c1)C(=O)\C=C\c2ccc(C)s2 | 4.51 | * | - | - | - | 72.48 | 69.28 | 77.43 | 47.91 | 4.69 | 4.51 | 4.39 | 4.36 |
| 54 | CCCCCOc1ccc(O)c(c1)C(=O)\C=C\c2ccc(C)s2 | 4.06 | - | + | + | - | 60.94 | 64.06 | 71.82 | 43.85 | 4.33 | 4.33 | 4.19 | 4.24 |
| 55 | COc1ccc(O)c(c1)C(=O)\C=C\c2ccc(C)s2 | 4.15 | + | # | + | # | 65.60 | 58.99 | 73.73 | 44.20 | 4.48 | 4.15 | 4.26 | 4.25 |
| 57 | CCCOc1ccc(OC)cc1C(=O)\C=C\c2ccc(C)s2 | 4.06 | * | # | * | # | 57.93 | 58.16 | 65.39 | 37.09 | 4.24 | 4.12 | 3.96 | 4.04 |
| 58 | CCCCOc1ccc(OC)cc1C(=O)\C=C\c2ccc(C)s2 | 4.21 | + | - | # | + | 59.74 | 59.94 | 67.12 | 39.03 | 4.29 | 4.19 | 4.03 | 4.10 |
| 59 | COc1ccc(C(=O)\C=C\c2ccc(C)s2)c(O)c1O | 4.27 | + | + | # | * | 59.32 | 56.40 | 72.92 | 42.14 | 4.28 | 4.06 | 4.23 | 4.19 |
| 60 | Cc1ccc(\C=C\C(=O)c2ccc(O)c(O)c2O)s1 | 4.45 | - | + | * | + | 55.47 | 65.91 | 74.43 | 49.61 | 4.16 | 4.40 | 4.29 | 4.41 |
| 61 | COc1ccc(\C=C\C(=O)c2cc(OC)c(OC)c(OC)c2)cc1OCC(=O)Nc3nc4ccccc4s3 | 5.82 | + | - | + | + | 92.31 | 94.63 | 106.52 | 79.03 | 5.30 | 5.40 | 5.43 | 5.27 |
| 62 | COc1ccc(\C=C\C(=O)c2cc(OC)c(OC)c(OC)c2)cc1OCC(=O)Nc3nc4ccc(F)cc4s3 | 4.92 | - | + | + | - | 90.91 | 93.80 | 106.30 | 81.66 | 5.26 | 5.38 | 5.42 | 5.34 |
| 63 | COc1ccc(\C=C\C(=O)c2cc(OC)c(OC)c(OC)c2)cc1OCC(=O)Nc3nc4ccc(Cl)cc4s3 | 5.22 | + | - | + | + | 101.94 | 97.45 | 106.45 | 85.77 | 5.60 | 5.50 | 5.42 | 5.46 |
| 64 | COc1ccc(\C=C\C(=O)c2cc(OC)c(OC)c(OC)c2)cc1OCC(=O)Nc3nc4ccc(cc4s3)N=O | 5.06 | - | - | - | * | 95.47 | 89.05 | 104.77 | 84.18 | 5.40 | 5.21 | 5.36 | 5.42 |
| 65 | COc1ccc2nc(NC(=O)COc3cc(\C=C\C(=O)c4cc(OC)c(OC)c(OC)c4)ccc3OC)sc2c1 | 5.47 | - | + | # | * | 97.49 | 92.98 | 113.05 | 90.89 | 5.46 | 5.35 | 5.66 | 5.61 |
| 66 | COc1ccc(\C=C\C(=O)c2cc(OC)c(OC)c(OC)c2)cc1OCC(=O)Nc3nc4ccc(OC(F)(F)F)cc4s3 | 5.96 | + | - | + | * | 107.79 | 110.71 | 115.97 | 91.43 | 5.78 | 5.97 | 5.76 | 5.63 |
| 67 | COc1ccc(\C=C\C(=O)c2cc(OC)c(OC)c(OC)c2)cc1OCC(=O)Nc3nc4ccc(cc4s3)C(F)(F)F | 5.43 | + | * | - | # | 102.49 | 105.35 | 110.09 | 91.45 | 5.62 | 5.78 | 5.55 | 5.63 |
| 68 | COc1ccc(\C=C\C(=O)c2cc(OC)c(OC)c(OC)c2)cc1OCC(=O)Nc3nc4ccc(C)cc4s3 | 5.68 | + | + | - | - | 93.24 | 91.56 | 104.23 | 80.17 | 5.33 | 5.30 | 5.34 | 5.30 |
| 69 | CCOc1ccc2nc(NC(=O)COc3cc(\C=C\C(=O)c4cc(OC)c(OC)c(OC)c4)ccc3OC)sc2c1 | 5.62 | + | + | - | + | 92.25 | 95.09 | 108.80 | 90.65 | 5.30 | 5.42 | 5.51 | 5.60 |
| 70 | COc1ccc(\C=C\C(=O)c2cc(OC)c(OC)c(OC)c2)cc1OCC(=O)Nc3nc4c(C)cccc4s3 | 5.22 | # | * | + | - | 91.98 | 93.52 | 101.48 | 82.81 | 5.29 | 5.37 | 5.25 | 5.38 |
| 71 | COc1ccc(\C=C\C(=O)c2cc(OC)c(OC)c(OC)c2)cc1OCC(=O)Nc3nc4cc(C)c(C)cc4s3 | 5.23 | # | * | * | + | 95.09 | 92.52 | 104.51 | 80.51 | 5.39 | 5.33 | 5.35 | 5.31 |
| 72 | COc1ccc(\C=C\C(=O)c2cc(OC)c(OC)c(OC)c2)cc1OCC(=O)Nc3ccc4ncsc4c3 | 5.08 | * | + | * | + | 88.69 | 93.73 | 102.96 | 77.94 | 5.19 | 5.37 | 5.30 | 5.23 |
| 73 | COc1ccc(\C=C\C(=O)c2cc(OC)c(OC)c(OC)c2)cc1OCC(=O)Nc3ccc4nc(C)sc4c3 | 5.02 | - | * | * | - | 82.47 | 82.37 | 99.12 | 70.90 | 5.00 | 4.97 | 5.16 | 5.03 |
| 75 | CC1(C)Cc2c(O1)ccc(C(=O)\C=C\c3ccc(F)cc3)c2OCc4ccccc4 | 4.64 | - | - | # | + | 71.52 | 77.03 | 84.48 | 60.36 | 4.66 | 4.79 | 4.64 | 4.72 |
| 76 | CC(=CCC[C@@]1(C)Oc2cc(O)c(cc2C=C1)C(=O)\C=C\c3ccc(O)cc3)C | 4.96 | + | # | - | # | 76.22 | 72.50 | 93.45 | 68.57 | 4.80 | 4.63 | 4.96 | 4.96 |
| 77 | CC(=CCC\C(=C\Cc1cc(C(=O)\C=C\c2ccc(O)cc2)c(O)cc1O)\C)C | 4.94 | - | # | # | * | 76.25 | 76.87 | 91.32 | 66.20 | 4.81 | 4.78 | 4.89 | 4.89 |
| 78 | CC(=CCC\C(=C\Cc1c(O)ccc(C(=O)CCc2ccc(O)c(O)c2)c1O)\C)C | 4.71 | # | * | + | + | 75.27 | 80.01 | 89.27 | 59.84 | 4.78 | 4.89 | 4.81 | 4.71 |
| 79 | CC(=CCC\C(=C\Cc1c(O)ccc(C(=O)\C=C\c2ccc(O)cc2)c1O)\C)C | 4.79 | - | + | # | # | 77.34 | 80.13 | 92.10 | 61.54 | 4.84 | 4.90 | 4.91 | 4.75 |
| 80 | Oc1ccc(O)c(c1)C(=O)\C=C\c2occc2 | 4.61 | # | - | + | * | 63.49 | 70.67 | 88.04 | 46.77 | 4.41 | 4.56 | 4.77 | 4.32 |
| 81 | Oc1ccc(O)c(c1)C(=O)\C=C\c2cocc2 | 4.67 | * | * | + | - | 61.70 | 65.56 | 89.14 | 51.88 | 4.35 | 4.38 | 4.81 | 4.47 |
| 82 | Oc1ccc(O)c(c1)C(=O)\C=C\c2cccs2 | 4.88 | - | + | * | - | 75.48 | 69.97 | 80.75 | 50.03 | 4.78 | 4.54 | 4.51 | 4.42 |
| 83 | Oc1ccc(O)c(c1)C(=O)\C=C\c2ccsc2 | 5.28 | + | - | - | - | 83.25 | 76.90 | 85.41 | 60.24 | 5.02 | 4.78 | 4.68 | 4.72 |
| 84 | Cc1oc(\C=C\C(=O)c2cc(O)ccc2O)cc1 | 4.37 | * | # | # | - | 58.04 | 65.31 | 78.96 | 51.09 | 4.24 | 4.37 | 4.45 | 4.45 |
| 86 | Cc1ccsc1\C=C\C(=O)c2cc(O)ccc2O | 4.71 | * | - | - | # | 71.66 | 71.19 | 80.58 | 56.61 | 4.66 | 4.58 | 4.50 | 4.61 |
| 87 | Oc1ccc(O)c(c1)C(=O)\C=C\c2oc(Br)cc2 | 4.82 | - | # | - | + | 63.02 | 64.84 | 83.10 | 65.35 | 4.40 | 4.36 | 4.59 | 4.87 |
| 88 | COc1ccc(OC)c(c1)C(=O)\C=C\c2ccc(OC\C=C\c3ccccc3)cc2 | 4.58 | - | + | + | * | 70.82 | 73.07 | 82.16 | 62.53 | 4.64 | 4.65 | 4.56 | 4.78 |
| 89 | COc1ccc(OC)c(c1)C(=O)\C=C\c2ccc(OC(=O)\C=C\c3ccc(C)cc3)cc2 | 4.79 | + | # | - | + | 76.14 | 84.23 | 88.86 | 65.41 | 4.80 | 5.04 | 4.80 | 4.87 |
| 90 | COc1ccc(\C=C\C(=O)Oc2ccc(\C=C\C(=O)c3cc(OC)ccc3OC)cc2)cc1 | 4.64 | + | # | + | # | 76.97 | 94.51 | 88.67 | 68.66 | 4.83 | 5.40 | 4.79 | 4.96 |
| 91 | COc1ccc(OC)c(c1)C(=O)\C=C\c2ccc(OC(=O)\C=C\c3cc(OC)c(OC)c(OC)c3)cc2 | 4.62 | - | # | # | # | 75.93 | 87.58 | 93.43 | 74.01 | 4.80 | 5.16 | 4.96 | 5.12 |
| 92 | COc1cc(cc(OC)c1OC)C2[C@@H]3[C@H](COC3=O)[C@H](NC(=O)CCCCCOc4ccc(\C=C\C(=O)c5cc(OC)c(OC)c(OC)c5)cc4)c6cc7OCOc7cc26 | 4.82 | + | * | * | - | 79.69 | 83.11 | 89.24 | 60.82 | 4.91 | 5.00 | 4.81 | 4.73 |
| 93 | COc1cccc(c1)C(=O)\C=C\c2ccc(OCCCCCC(=O)N[C@H]3[C@H]4COC(=O)[C@@H]4C(c5cc(OC)c(OC)c(OC)c5)c6cc7OCOc7cc36)c(OC)c2 | 4.92 | # | + | - | # | 81.64 | 79.32 | 92.10 | 71.95 | 4.97 | 4.87 | 4.91 | 5.06 |
| 94 | COc1ccc(cc1)C(=O)\C=C\c2ccc(OCCCCCC(=O)N[C@H]3[C@H]4COC(=O)[C@@H]4C(c5cc(OC)c(OC)c(OC)c5)c6cc7OCOc7cc36)c(OC)c2 | 4.79 | - | - | + | # | 84.47 | 82.29 | 92.57 | 73.13 | 5.06 | 4.97 | 4.93 | 5.09 |
| 95 | COc1cc(\C=C\C(=O)c2cccc(c2)N=O)ccc1OCCCCCC(=O)N[C@H]3[C@H]4COC(=O)[C@@H]4C(c5cc(OC)c(OC)c(OC)c5)c6cc7OCOc7cc36 | 5.06 | - | # | # | - | 82.90 | 87.09 | 90.26 | 71.91 | 5.01 | 5.14 | 4.85 | 5.06 |
| 96 | COc1cc(\C=C\C(=O)c2cc(OC)c(OC)c(OC)c2)ccc1OCCCCCC(=O)N[C@H]3[C@H]4COC(=O)[C@@H]4C(c5cc(OC)c(OC)c(OC)c5)c6cc7OCOc7cc36 | 4.92 | + | + | * | - | 81.70 | 84.24 | 91.44 | 67.70 | 4.97 | 5.04 | 4.89 | 4.93 |
| 97 | COc1cc(\C=C\C(=O)c2ccc(F)cc2)ccc1OCCCCCC(=O)N[C@H]3[C@H]4COC(=O)[C@@H]4C(c5cc(OC)c(OC)c(OC)c5)c6cc7OCOc7cc36 | 4.93 | # | + | * | * | 78.83 | 82.87 | 91.12 | 66.24 | 4.89 | 4.99 | 4.88 | 4.89 |
| 98 | COc1cc(cc(OC)c1OC)C2[C@@H]3[C@H](COC3=O)[C@H](NC(=O)CCCCCOc4cccc(\C=C\C(=O)c5cc(OC)c(OC)c(OC)c5)c4)c6cc7OCOc7cc26 | 4.68 | * | - | + | - | 77.84 | 79.73 | 88.96 | 60.99 | 4.85 | 4.88 | 4.80 | 4.74 |
| 99 | COc1ccc(\C=C\C(=O)c2cc(OC)c(OC)c(OC)c2)cc1OCCCCCC(=O)N[C@H]3[C@H]4COC(=O)[C@@H]4C(c5cc(OC)c(OC)c(OC)c5)c6cc7OCOc7cc36 | 5.19 | - | - | - | + | 81.06 | 86.46 | 91.35 | 66.77 | 4.95 | 5.12 | 4.89 | 4.91 |
| 100 | COc1cc(cc(OC)c1OC)C2[C@@H]3[C@H](COC3=O)[C@H](NC(=O)COc4ccc(\C=C\C(=O)c5cc(OC)c(OC)c(OC)c5)cc4)c6cc7OCOc7cc26 | 4.91 | + | * | + | * | 78.16 | 77.46 | 87.31 | 60.52 | 4.86 | 4.80 | 4.74 | 4.73 |
| 101 | COc1cccc(c1)C(=O)\C=C\c2ccc(OCC(=O)N[C@H]3[C@H]4COC(=O)[C@@H]4C(c5cc(OC)c(OC)c(OC)c5)c6cc7OCOc7cc36)c(OC)c2 | 5.09 | * | # | - | - | 77.44 | 73.99 | 92.28 | 73.12 | 4.84 | 4.68 | 4.92 | 5.09 |
| 102 | COc1ccc(cc1)C(=O)\C=C\c2ccc(OCC(=O)N[C@H]3[C@H]4COC(=O)[C@@H]4C(c5cc(OC)c(OC)c(OC)c5)c6cc7OCOc7cc36)c(OC)c2 | 4.77 | # | + | - | * | 80.27 | 76.96 | 92.75 | 74.30 | 4.93 | 4.78 | 4.94 | 5.13 |
| 103 | COc1cc(\C=C\C(=O)c2cccc(c2)N=O)ccc1OCC(=O)N[C@H]3[C@H]4COC(=O)[C@@H]4C(c5cc(OC)c(OC)c(OC)c5)c6cc7OCOc7cc36 | 5.08 | # | * | - | + | 78.69 | 81.76 | 90.44 | 73.08 | 4.88 | 4.95 | 4.85 | 5.09 |
| 104 | COc1cc(\C=C\C(=O)c2cc(OC)c(OC)c(OC)c2)ccc1OCC(=O)N[C@H]3[C@H]4COC(=O)[C@@H]4C(c5cc(OC)c(OC)c(OC)c5)c6cc7OCOc7cc36 | 5.02 | * | * | + | + | 77.50 | 78.91 | 91.62 | 68.87 | 4.84 | 4.85 | 4.90 | 4.97 |
| 105 | COc1cc(\C=C\C(=O)c2ccc(F)cc2)ccc1OCC(=O)N[C@H]3[C@H]4COC(=O)[C@@H]4C(c5cc(OC)c(OC)c(OC)c5)c6cc7OCOc7cc36 | 4.67 | + | - | - | + | 74.62 | 77.53 | 91.31 | 67.41 | 4.75 | 4.80 | 4.88 | 4.93 |
| 106 | COc1cc(cc(OC)c1OC)C2[C@@H]3[C@H](COC3=O)[C@H](NC(=O)COC4=CC(CC=C4)\C=C\C(=O)c5cc(OC)c(OC)c(OC)c5)c6cc7OCOc7cc26 | 4.81 | * | # | # | - | 92.51 | 80.67 | 91.12 | 69.49 | 5.31 | 4.91 | 4.88 | 4.99 |
| 107 | COC1=CCC(\C=C\C(=O)c2cc(OC)c(OC)c(OC)c2)C=C1OCC(=O)N[C@H]3[C@H]4COC(=O)[C@@H]4C(c5cc(OC)c(OC)c(OC)c5)c6cc7OCOc7cc36 | 5.22 | + | - | * | # | 88.05 | 87.95 | 101.17 | 72.46 | 5.17 | 5.17 | 5.24 | 5.07 |
| 108 | COc1cc(cc(OC)c1OC)C2[C@@H]3[C@H](COC3=O)[C@H](NC(=O)COc4c(C)cc(\C=C\C(=O)c5c(C)nc6ccccc6c5C)cc4OC)c7cc8OCOc8cc27 | 4.91 | + | - | - | # | 75.48 | 80.94 | 93.25 | 73.01 | 4.78 | 4.92 | 4.95 | 5.09 |
| 109 | COc1cc(cc(OC)c1OC)C2[C@@H]3[C@H](COC3=O)[C@H](NC(=O)COc4c(C)cc(\C=C\C(=O)c5c(C)nc6ccccc6c5c7ccccc7)cc4OC)c8cc9OCOc9cc28~ | 5.11 | # | # | # | + | 86.56 | 86.26 | 95.94 | 72.50 | 5.13 | 5.11 | 5.05 | 5.07 |
| 110 | COc1cc(\C=C\C(=O)c2c(C)nc3ccccc3c2C)ccc1OCCCCCC(=O)N[C@H]4C5COC(=O)C5C(c6cc(OC)c(OC)c(OC)c6)c7cc8OCOc8cc47 | 4.93 | - | + | * | * | 78.64 | 78.26 | 92.84 | 67.85 | 4.88 | 4.83 | 4.94 | 4.94 |
| 111 | COc1cc(\C=C\C(=O)c2c(C)nc3ccccc3c2Cc4ccccc4)ccc1OCCCCCC(=O)N[C@H]5C6COC(=O)C6C(c7cc(OC)c(OC)c(OC)c7)c8cc9OCOc9cc58 | 5.05 | - | - | # | * | 92.13 | 94.87 | 99.15 | 70.45 | 5.30 | 5.41 | 5.16 | 5.02 |
| 113 | COc1cc(cc(OC)c1OC)C2C3C(COC3=O)[C@H](NC(=O)\C=C\c4ccc(F)cc4)c5cc6OCOc6cc25 | 5.15 | * | * | + | # | 75.28 | 76.18 | 92.97 | 57.12 | 4.78 | 4.76 | 4.94 | 4.63 |
| 114 | COc1cc(cc(OC)c1OC)C2C3C(COC3=O)[C@H](NC(=O)\C=C\c4ccc(Cl)cc4)c5cc6OCOc6cc25 | 4.94 | * | + | - | + | 88.46 | 81.85 | 96.81 | 64.63 | 5.18 | 4.96 | 5.08 | 4.85 |
| 115 | COc1cc(cc(OC)c1OC)C2C3C(COC3=O)[C@H](NC(=O)\C=C\c4ccc(Br)cc4)c5cc6OCOc6cc25 | 4.72 | * | # | # | + | 82.25 | 75.17 | 89.34 | 62.79 | 4.99 | 4.72 | 4.81 | 4.79 |
| 116 | COc1ccc(\C=C\C(=O)N[C@H]2C3COC(=O)C3C(c4cc(OC)c(OC)c(OC)c4)c5cc6OCOc6cc25)cc1 | 4.75 | + | * | + | * | 80.28 | 79.03 | 88.20 | 57.97 | 4.93 | 4.86 | 4.77 | 4.65 |
| 118 | COc1cc(cc(OC)c1OC)C2C3C(COC3=O)[C@H](NC(=O)\C=C\c4ccc(cc4)N=O)c5cc6OCOc6cc25 | 4.74 | # | + | - | # | 80.10 | 74.37 | 88.30 | 62.49 | 4.92 | 4.69 | 4.78 | 4.78 |
| 119 | COc1cc(cc(OC)c1OC)C2C3C(COC3=O)[C@H](NC(=O)\C=C\c4ccc(Cl)c(Cl)c4)c5cc6OCOc6cc25 | 4.68 | # | - | # | # | 92.35 | 87.05 | 97.28 | 70.57 | 5.30 | 5.14 | 5.10 | 5.02 |
| 120 | COc1cc(\C=C\C(=O)N[C@H]2C3COC(=O)C3C(c4cc(OC)c(OC)c(OC)c4)c5cc6OCOc6cc25)cc(OC)c1OC | 5.08 | - | + | + | # | 76.05 | 76.29 | 91.39 | 61.98 | 4.80 | 4.76 | 4.89 | 4.77 |
| 121 | COc1ccc(C(=O)\C=C\c2ccc(cc2)N(C)C)c3OC(C)(C)C=Cc13 | 5.73 | + | - | + | + | 97.14 | 92.96 | 101.30 | 81.58 | 5.45 | 5.35 | 5.24 | 5.34 |
| 122 | COc1ccc(C(=O)\C=C\c2ccc3ccccc3c2)c4OC(C)(C)C=Cc14 | 5.35 | # | # | + | + | 95.45 | 86.94 | 105.81 | 67.61 | 5.40 | 5.13 | 5.40 | 4.93 |
| 124 | COc1ccc(C(=O)\C=C\c2ccc(cc2)N3CCCC3)c4OC(C)(C)C=Cc14 | 5.08 | * | + | - | * | 98.14 | 93.66 | 101.07 | 74.60 | 5.48 | 5.37 | 5.23 | 5.14 |
| 125 | COc1ccc(C(=O)\C=C\c2ccc(cc2)n3ccnc3)c4OC(C)(C)C=Cc14 | 5.18 | - | + | * | + | 92.14 | 85.20 | 101.23 | 84.31 | 5.30 | 5.07 | 5.24 | 5.42 |
| 126 | COc1ccc(C(=O)\C=C\c2ccc3ccccc3n2)c4OC(C)(C)C=Cc14 | 5.42 | + | - | - | * | 102.16 | 94.18 | 110.30 | 79.57 | 5.61 | 5.39 | 5.56 | 5.28 |
| 127 | COc1ccc(C(=O)\C=C\c2ccc(cc2)[N+](=O)[O-])c3OC(C)(C)C=Cc13 | 5.43 | # | # | # | * | 96.56 | 90.86 | 99.78 | 91.00 | 5.44 | 5.27 | 5.19 | 5.61 |
| 128 | COc1ccc(C(=O)\C=C\c2cccc(c2)[N+](=O)[O-])c3OC(C)(C)C=Cc13 | 5.37 | + | + | # | + | 94.83 | 93.54 | 100.10 | 88.35 | 5.38 | 5.37 | 5.20 | 5.54 |
| 129 | COc1ccc(C(=O)\C=C\c2cccc(c2)C(F)(F)F)c3OC(C)(C)C=Cc13 | 5.04 | - | + | + | + | 96.80 | 99.00 | 102.74 | 81.36 | 5.44 | 5.56 | 5.29 | 5.33 |
| 130 | COc1ccc(C(=O)\C=C\c2ccccn2)c3OC(C)(C)C=Cc13 | 5.67 | - | + | - | - | 92.64 | 92.42 | 109.08 | 81.22 | 5.31 | 5.33 | 5.52 | 5.33 |
| 131 | COc1ccc(C(=O)\C=C\c2ccc(Cl)c(Cl)c2)c3OC(C)(C)C=Cc13 | 5.12 | # | - | + | - | 101.68 | 101.71 | 100.72 | 82.33 | 5.59 | 5.65 | 5.22 | 5.36 |
| 132 | COc1ccc(C(=O)\C=C\c2cc(Cl)ccc2O)c3OC(C)(C)C=Cc13 | 5.3 | - | - | # | # | 97.19 | 97.09 | 98.35 | 82.96 | 5.45 | 5.49 | 5.14 | 5.38 |
| 133 | C[C@@H]1CC[C@H]2[C@@H](C)[C@@H](NC(=O)COc3ccc(cc3)C(=O)\C=C\c4ccc(F)cc4F)O[C@]56OO[C@](C)(CC[C@@H]1C25)O6 | 6.03 | * | * | + | * | 115.23 | 123.50 | 122.74 | 115.38 | 6.01 | 6.42 | 6.00 | 6.33 |
| 134 | COc1cc(\C=C\C(=O)c2ccc(OCC(=O)N[C@H]3O[C@]45OO[C@](C)(CC[C@H]6[C@H](C)CC[C@@H]([C@H]3C)C46)O5)cc2)cc(OC)c1OC | 6.62 | # | - | - | + | 120.66 | 129.05 | 136.01 | 124.11 | 6.18 | 6.61 | 6.47 | 6.58 |
| 135 | C[C@@H]1CC[C@H]2[C@@H](C)[C@@H](NC(=O)COc3ccc(cc3)C(=O)\C=C\c4ccc(OC(F)(F)F)cc4)O[C@]56OO[C@](C)(CC[C@@H]1C25)O6 | 6.44 | + | - | + | - | 132.70 | 135.16 | 137.13 | 123.96 | 6.56 | 6.83 | 6.51 | 6.58 |
| 136 | C[C@@H]1CC[C@H]2[C@@H](C)[C@@H](NC(=O)COc3ccc(cc3)C(=O)\C=C\c4cccc(c4)C(F)(F)F)O[C@]56OO[C@](C)(CC[C@@H]1C25)O6 | 6.59 | * | * | # | * | 129.38 | 129.11 | 128.05 | 122.33 | 6.45 | 6.62 | 6.19 | 6.53 |
| 137 | C[C@@H]1CC[C@H]2[C@@H](C)[C@@H](NC(=O)COc3ccc(cc3)C(=O)\C=C\c4ccc(F)cc4)O[C@]56OO[C@](C)(CC[C@@H]1C25)O6 | 6.16 | # | - | # | - | 115.44 | 116.43 | 126.48 | 108.95 | 6.02 | 6.17 | 6.13 | 6.14 |
| 138 | C[C@@H]1CC[C@H]2[C@@H](C)[C@@H](NC(=O)COc3ccc(cc3)C(=O)\C=C\c4ccc(Cl)cc4)O[C@]56OO[C@](C)(CC[C@@H]1C25)O6 | 6.3 | # | + | + | # | 128.63 | 122.11 | 132.89 | 116.45 | 6.43 | 6.37 | 6.36 | 6.36 |
| 139 | C[C@@H]1CC[C@H]2[C@@H](C)[C@@H](NC(=O)COc3ccc(cc3)C(=O)\C=C\c4c(Cl)cccc4Cl)O[C@]56OO[C@](C)(CC[C@@H]1C25)O6 | 6.85 | - | + | - | # | 131.71 | 128.01 | 134.22 | 117.06 | 6.53 | 6.58 | 6.41 | 6.37 |
| 140 | COc1cccc(\C=C\C(=O)c2ccc(OCC(=O)N[C@H]3O[C@]45OO[C@](C)(CC[C@H]6[C@H](C)CC[C@@H]([C@H]3C)C46)O5)cc2)c1 | 6.14 | * | - | + | * | 123.99 | 129.67 | 132.67 | 121.10 | 6.29 | 6.64 | 6.35 | 6.49 |
| 141 | C[C@@H]1CC[C@H]2[C@@H](C)[C@@H](NC(=O)COc3ccc(cc3)C(=O)\C=C\c4ccccc4)O[C@]56OO[C@](C)(CC[C@@H]1C25)O6 | 6.19 | + | + | * | + | 117.05 | 112.31 | 123.38 | 106.83 | 6.07 | 6.03 | 6.02 | 6.08 |
| 142 | C[C@@H]1CC[C@H]2[C@@H](C)[C@@H](NC(=O)COc3ccc(cc3)C(=O)\C=C\c4occc4)O[C@]56OO[C@](C)(CC[C@@H]1C25)O6 | 5.64 | + | # | # | + | 109.49 | 106.83 | 118.02 | 100.41 | 5.84 | 5.83 | 5.83 | 5.89 |
| 143 | C[C@@H]1CC[C@H]2[C@@H](C)[C@@H](NC(=O)COc3ccc(cc3)C(=O)\C=C\c4cccs4)O[C@]56OO[C@](C)(CC[C@@H]1C25)O6 | 5.8 | # | - | # | - | 116.46 | 116.65 | 124.66 | 113.68 | 6.05 | 6.18 | 6.07 | 6.28 |
| 144 | C[C@@H]1CC[C@H]2[C@@H](C)[C@@H](NC(=O)COc3ccc(cc3)C(=O)\C=C\c4ccc5OCOc5c4)O[C@]67OO[C@](C)(CC[C@@H]1C26)O7 | 5.92 | - | + | - | * | 111.60 | 108.14 | 116.07 | 97.02 | 5.90 | 5.88 | 5.76 | 5.79 |
| 145 | C[C@@H]1CC[C@H]2[C@@H](C)[C@H](NC(=O)COc3ccc(cc3)C(=O)\C=C\c4ccc(F)cc4F)O[C@]56OO[C@](C)(CC[C@@H]1C25)O6 | 5.59 | - | # | - | # | 113.25 | 124.41 | 122.39 | 117.72 | 5.95 | 6.45 | 5.99 | 6.39 |
| 146 | COc1cc(\C=C\C(=O)c2ccc(OCC(=O)N[C@@H]3O[C@]45OO[C@](C)(CC[C@H]6[C@H](C)CC[C@@H]([C@H]3C)C46)O5)cc2)cc(OC)c1OC | 6.16 | + | # | - | - | 122.63 | 128.13 | 136.36 | 121.77 | 6.24 | 6.58 | 6.49 | 6.51 |
| 147 | C[C@@H]1CC[C@H]2[C@@H](C)[C@H](NC(=O)COc3ccc(cc3)C(=O)\C=C\c4ccc(OC(F)(F)F)cc4)O[C@]56OO[C@](C)(CC[C@@H]1C25)O6 | 6.41 | - | # | # | * | 130.73 | 136.07 | 136.79 | 126.30 | 6.50 | 6.86 | 6.50 | 6.64 |
| 148 | C[C@@H]1CC[C@H]2[C@@H](C)[C@H](NC(=O)COc3ccc(cc3)C(=O)\C=C\c4cccc(c4)C(F)(F)F)O[C@]56OO[C@](C)(CC[C@@H]1C25)O6 | 6.62 | - | # | # | - | 127.41 | 130.02 | 127.71 | 124.67 | 6.39 | 6.65 | 6.18 | 6.60 |
| 149 | C[C@@H]1CC[C@H]2[C@@H](C)[C@H](NC(=O)COc3ccc(cc3)C(=O)\C=C\c4ccc(F)cc4)O[C@]56OO[C@](C)(CC[C@@H]1C25)O6 | 6.12 | + | # | + | # | 113.47 | 117.35 | 126.13 | 111.28 | 5.96 | 6.20 | 6.12 | 6.21 |
| 150 | C[C@@H]1CC[C@H]2[C@@H](C)[C@H](NC(=O)COc3ccc(cc3)C(=O)\C=C\c4ccc(Cl)cc4)O[C@]56OO[C@](C)(CC[C@@H]1C25)O6 | 6.17 | + | - | # | * | 126.65 | 123.02 | 132.54 | 118.79 | 6.37 | 6.40 | 6.35 | 6.43 |
| 151 | C[C@@H]1CC[C@H]2[C@@H](C)[C@H](NC(=O)COc3ccc(cc3)C(=O)\C=C\c4c(Cl)cccc4Cl)O[C@]56OO[C@](C)(CC[C@@H]1C25)O6 | 6.3 | - | + | - | - | 129.73 | 128.92 | 133.88 | 119.40 | 6.46 | 6.61 | 6.40 | 6.44 |
| 152 | COc1cccc(\C=C\C(=O)c2ccc(OCC(=O)N[C@@H]3O[C@]45OO[C@](C)(CC[C@H]6[C@H](C)CC[C@@H]([C@H]3C)C46)O5)cc2)c1 | 6.17 | + | - | * | # | 125.96 | 128.76 | 133.02 | 118.76 | 6.35 | 6.60 | 6.37 | 6.42 |
| 153 | C[C@@H]1CC[C@H]2[C@@H](C)[C@H](NC(=O)COc3ccc(cc3)C(=O)\C=C\c4ccccc4)O[C@]56OO[C@](C)(CC[C@@H]1C25)O6 | 6.13 | - | # | - | - | 115.08 | 113.22 | 123.03 | 109.17 | 6.01 | 6.06 | 6.01 | 6.14 |
| 154 | C[C@@H]1CC[C@H]2[C@@H](C)[C@H](NC(=O)COc3ccc(cc3)C(=O)\C=C\c4occc4)O[C@]56OO[C@](C)(CC[C@@H]1C25)O6 | 5.85 | * | + | + | # | 107.51 | 107.75 | 117.67 | 102.75 | 5.78 | 5.87 | 5.82 | 5.96 |
| 155 | C[C@@H]1CC[C@H]2[C@@H](C)[C@H](NC(=O)COc3ccc(cc3)C(=O)\C=C\c4cccs4)O[C@]56OO[C@](C)(CC[C@@H]1C25)O6 | 5.68 | - | - | - | - | 114.49 | 117.56 | 124.31 | 116.02 | 5.99 | 6.21 | 6.06 | 6.34 |
| 156 | C[C@@H]1CC[C@H]2[C@@H](C)[C@H](NC(=O)COc3ccc(cc3)C(=O)\C=C\c4ccc5OCOc5c4)O[C@]67OO[C@](C)(CC[C@@H]1C26)O7 | 5.77 | - | + | # | # | 109.62 | 109.05 | 115.72 | 99.36 | 5.84 | 5.91 | 5.75 | 5.86 |
| 157 | COc1cc(O)c(C(=O)CCc2ccc3OCOc3c2)c(OC)c1 | 3.84 | + | - | + | + | 52.15 | 65.01 | 67.37 | 41.82 | 4.06 | 4.36 | 4.03 | 4.18 |
| 158 | COc1cccc(c1)C(=C)C(=C)C(=O)c2c(O)cc(OC)cc2OC | 4.34 | # | * | # | - | 66.34 | 69.83 | 83.08 | 49.16 | 4.50 | 4.53 | 4.59 | 4.39 |
| 159 | COc1cccc(c1)C(=C)C(=C)C(=O)c2c(O)cc(O)cc2OC | 4.67 | - | + | + | + | 66.10 | 68.73 | 85.07 | 54.07 | 4.49 | 4.49 | 4.66 | 4.54 |
| 160 | COc1cc(O)cc(O)c1C(=O)C(=C)C(=C)c2ccc3OCOc3c2 | 4.68 | - | - | - | + | 59.66 | 71.71 | 82.55 | 52.39 | 4.29 | 4.60 | 4.57 | 4.49 |
| 161 | COc1cccc(c1)C(=C)C(=C)C(=O)c2c(OC)cc(OCC=C)cc2OCC=C | 4.55 | - | # | + | * | 69.79 | 71.48 | 78.07 | 53.98 | 4.61 | 4.59 | 4.41 | 4.53 |
| 162 | COc1cc(cc(OC)c1OC)c2nc(\C=C\C(=O)c3ccc(cc3)[N+](=O)[O-])cc4c5ccccc5[nH]c24 | 4.32 | # | - | - | * | 60.71 | 58.98 | 74.02 | 65.51 | 4.32 | 4.15 | 4.27 | 4.87 |
| 163 | COc1cc(cc(OC)c1OC)c2nc(\C=C\C(=O)c3ccc(N)cc3)cc4c5ccccc5[nH]c24 | 4.26 | + | - | # | # | 57.30 | 56.37 | 70.61 | 47.00 | 4.22 | 4.06 | 4.15 | 4.33 |
| 164 | COc1ccc(cc1)C(=O)\C=C\c2cc3c4ccccc4[nH]c3c(n2)c5ccc(F)c(OC)c5 | 4.22 | - | - | + | + | 59.49 | 66.48 | 78.63 | 42.19 | 4.29 | 4.42 | 4.43 | 4.19 |
| 165 | COc1ccc2c(c1)sc3nc(\C=C\C(=O)c4cc(OC)c(OC)c(OC)c4)cn23 | 4.69 | # | # | # | # | 75.03 | 72.00 | 89.65 | 55.13 | 4.77 | 4.61 | 4.83 | 4.57 |
| 166 | COc1ccc2c(c1)sc3nc(\C=C\C(=O)c4cc(OC)cc(OC)c4)cn23 | 4.91 | * | + | + | - | 72.65 | 74.90 | 87.83 | 57.77 | 4.69 | 4.71 | 4.76 | 4.64 |
| 167 | COc1ccc2c(c1)sc3nc(\C=C\C(=O)c4ccc(OC)c(OC)c4)cn23 | 4.59 | * | # | # | - | 75.68 | 71.75 | 87.84 | 60.00 | 4.79 | 4.60 | 4.76 | 4.71 |
| 168 | COc1ccc2c(c1)sc3nc(\C=C\C(=O)c4ccc(OC)c(O)c4)cn23 | 4.92 | # | # | + | - | 74.74 | 70.89 | 90.71 | 64.23 | 4.76 | 4.57 | 4.86 | 4.83 |
| 169 | COc1ccc2c(c1)sc3nc(\C=C\C(=O)c4cc(OC)c(OC)c(OC)c4Br)cn23 | 4.46 | * | - | # | - | 77.49 | 77.16 | 88.05 | 62.08 | 4.84 | 4.79 | 4.77 | 4.77 |
| 170 | CCOc1ccc2c(c1)sc3nc(\C=C\C(=O)c4cc(OC)c(OC)c(OC)c4)cn23 | 4.42 | + | + | * | - | 69.79 | 74.11 | 85.40 | 54.89 | 4.61 | 4.68 | 4.68 | 4.56 |
| 171 | CCOc1ccc2c(c1)sc3nc(\C=C\C(=O)c4cc(OC)cc(OC)c4)cn23 | 4.54 | + | - | - | # | 67.41 | 77.01 | 83.58 | 57.53 | 4.53 | 4.79 | 4.61 | 4.64 |
| 172 | CCOc1ccc2c(c1)sc3nc(\C=C\C(=O)c4ccc(OC)c(OC)c4)cn23 | 4.47 | - | # | + | + | 70.43 | 73.86 | 83.59 | 59.76 | 4.63 | 4.67 | 4.61 | 4.70 |
| 173 | CCOc1ccc2c(c1)sc3nc(\C=C\C(=O)c4ccc(OC)c(O)c4)cn23 | 4.93 | * | * | - | + | 69.50 | 73.00 | 86.47 | 63.99 | 4.60 | 4.64 | 4.71 | 4.83 |
| 174 | CCOc1ccc2c(c1)sc3nc(\C=C\C(=O)c4ccc(OC)cc4)cn23 | 4.91 | # | + | # | + | 73.01 | 77.51 | 86.11 | 58.21 | 4.70 | 4.80 | 4.70 | 4.66 |
| 175 | CCOc1ccc2c(c1)sc3nc(\C=C\C(=O)c4cc(OC)c(OC)c(OC)c4Br)cn23 | 4.69 | # | # | - | # | 72.24 | 79.27 | 83.80 | 61.84 | 4.68 | 4.86 | 4.62 | 4.76 |
| 176 | COc1cc(cc(OC)c1OC)C(=O)\C=C\c2cn3c4ccc(C)cc4sc3n2 | 4.74 | + | + | - | + | 83.62 | 83.68 | 92.50 | 65.43 | 5.03 | 5.02 | 4.93 | 4.87 |
| 177 | COc1cc(OC)cc(c1)C(=O)\C=C\c2cn3c4ccc(C)cc4sc3n2 | 4.56 | + | - | - | + | 79.77 | 83.73 | 94.20 | 68.35 | 4.91 | 5.02 | 4.99 | 4.95 |
| 178 | COc1ccc(cc1OC)C(=O)\C=C\c2cn3c4ccc(C)cc4sc3n2 | 5.02 | - | - | * | * | 87.65 | 83.20 | 93.47 | 67.82 | 5.16 | 5.00 | 4.96 | 4.94 |
| 179 | COc1ccc(cc1)C(=O)\C=C\c2cn3c4ccc(C)cc4sc3n2 | 4.97 | # | - | * | # | 81.55 | 83.17 | 93.77 | 68.23 | 4.97 | 5.00 | 4.97 | 4.95 |
| 180 | COc1cc(C(=O)\C=C\c2cn3c4ccc(C)cc4sc3n2)c(Br)c(OC)c1OC | 4.67 | + | # | + | * | 83.79 | 82.41 | 90.28 | 75.37 | 5.04 | 4.97 | 4.85 | 5.16 |
| 181 | COc1cc(cc(OC)c1OC)C(=O)\C=C\c2cn3c4ccccc4sc3n2 | 4.61 | * | + | # | - | 83.53 | 84.10 | 94.31 | 67.18 | 5.03 | 5.03 | 4.99 | 4.92 |
| 182 | COc1cc(OC)cc(c1)C(=O)\C=C\c2cn3c4ccccc4sc3n2 | 4.61 | - | - | + | - | 79.68 | 84.15 | 96.01 | 70.10 | 4.91 | 5.04 | 5.05 | 5.00 |
| 183 | COc1ccc(cc1OC)C(=O)\C=C\c2cn3c4ccccc4sc3n2 | 5.35 | + | + | * | * | 87.55 | 83.62 | 95.28 | 69.57 | 5.16 | 5.02 | 5.03 | 4.99 |
| 184 | COc1ccc(cc1O)C(=O)\C=C\c2cn3c4ccccc4sc3n2 | 5.15 | - | - | + | + | 81.69 | 82.42 | 96.75 | 73.02 | 4.97 | 4.98 | 5.08 | 5.09 |
| 185 | COc1ccc(cc1)C(=O)\C=C\c2cn3c4ccccc4sc3n2 | 5.66 | + | + | + | - | 81.45 | 83.60 | 95.58 | 69.98 | 4.97 | 5.02 | 5.04 | 5.00 |
| 186 | COc1cc(C(=O)\C=C\c2cn3c4ccccc4sc3n2)c(Br)c(OC)c1OC | 5.04 | # | # | + | # | 83.70 | 82.83 | 92.09 | 77.12 | 5.04 | 4.99 | 4.91 | 5.21 |
| 187 | COc1cc(cc(OC)c1OC)C(=O)\C=C\c2cn3c4ccc(F)cc4sc3n2 | 5.13 | + | + | * | * | 82.45 | 80.58 | 95.64 | 66.89 | 5.00 | 4.91 | 5.04 | 4.91 |
| 188 | COc1cc(OC)cc(c1)C(=O)\C=C\c2cn3c4ccc(F)cc4sc3n2 | 4.97 | * | - | - | - | 78.60 | 80.63 | 97.35 | 69.81 | 4.88 | 4.91 | 5.10 | 5.00 |
| 189 | COc1ccc(cc1OC)C(=O)\C=C\c2cn3c4ccc(F)cc4sc3n2 | 4.81 | - | - | # | # | 86.47 | 80.10 | 96.61 | 69.27 | 5.12 | 4.89 | 5.07 | 4.98 |
| 190 | COc1ccc(cc1)C(=O)\C=C\c2cn3c4ccc(F)cc4sc3n2 | 5.32 | + | * | + | + | 80.37 | 80.07 | 96.92 | 69.68 | 4.93 | 4.89 | 5.08 | 4.99 |
| 191 | COc1cc(C(=O)\C=C\c2cn3c4ccc(F)cc4sc3n2)c(Br)c(OC)c1OC | 5.09 | # | # | * | + | 81.82 | 83.01 | 92.55 | 74.17 | 4.98 | 5.00 | 4.93 | 5.12 |
| 192 | COc1cc(OCC=C)cc(OCC=C)c1C(=O)C(=C)C(=C)c2ccc3OCOc3c2 | 4.62 | * | * | - | * | 65.84 | 77.10 | 77.35 | 53.24 | 4.48 | 4.79 | 4.39 | 4.51 |
| 193 | COc1cc(cc(OC)c1OC)C(=O)\C=C\c2cc3c4ccccc4[nH]c3c(n2)c5cc(OC)c(OC)c(OC)c5 | 4.64 | * | * | + | * | 63.34 | 66.85 | 79.54 | 40.08 | 4.41 | 4.43 | 4.47 | 4.13 |
