# Supplementary material for: SMILES-based QSAR and molecular docking studies of chalcone analogues as potential anti-colon cancer
Source: Sci Rep. 2025 Feb 24;15:6573. doi: 10.1038/s41598-025-91338-9 (PMC11850874; doi:10.1038/s41598-025-91338-9)
Supplement: Supplementary file 2 — Supplementary Material 2 [file 41598_2025_91338_MOESM2_ESM.docx]

**Table S1**. The molecular structures of chalcone derivatives are used in the present study.

|  | | | | | | | | | |
| --- | --- | --- | --- | --- | --- | --- | --- | --- | --- |
| Reference | pIC_50_ exp. | R_6_ | R_5_ | R_4_ | R_3_ | R_2_ | R_1_ | R | No. |
| [1] | 4.40 | Cl | H | Cl | H | NH_2_ | H | H | 1 |
| [1] | 4.72 | Cl | H | Cl | H | OCH_3_ | H | H | 2 |
| [1] | 4.37 | Cl | H | Cl | H | H | H | OH | 3 |
| [1] | 4.53 | H | H | Cl | H | NH_2_ | H | H | 4 |
| [1] | 4.62 | OCH_3_ | H | H | H | H | H | H | 5 |
| [1] | 4.25 | OCH_3_ | H | H | H | NH_2_ | H | H | 6 |
| [1] | 4.37 | OCH_3_ | H | H | H | H | NH_2_ | H | 7 |
| [1] | 4.31 | OCH_3_ | H | H | H | OCH_3_ | H | H | 8 |
| [1] | 4.23 | OCH_3_ | H | H | H | H | H | OH | 9 |
| [1] | 4.00 | OCH_3_ | H | H | F | H | F | H | 10 |
| [1] | 4.32 | H | H | F | H | NH_2_ | H | H | 11 |
| [1] | 4.72 | H | H | F | H | OCH_3_ | H | H | 12 |
| [1] | 4.61 | OH | H | H | H | H | H | H | 13 |
| [1] | 4.73 | OH | H | H | H | OCH_3_ | H | H | 14 |
| [1] | 3.88 | OCH_3_ | H | OCH_3_ | H | H | H | H | 15 |
| [1] | 4.27 | H | OCH_3_ | H | F | F | H | H | 16 |
| [1] | 4.16 | H | OCH_3_ | OCH_3_ | H | NH_2_ | H | H | 17 |
| [1] | 4.36 | H | OCH_3_ | OCH_3_ | H | H | NH_2_ | H | 18 |
| [1] | 4.38 | H | OCH_3_ | OCH_3_ | H | OCH_3_ | H | H | 19 |
| [1] | 4.37 | H | H | CF_3_ | H | H | H | H | 20 |
| [1] | 4.44 | H | H | CF_3_ | H | OCH_3_ | H | H | 21 |
| [1] | 4.45 | H | H | Br | H | NH_2_ | H | H | 22 |
| [1] | 4.59 | H | H | Br | H | OCH_3_ | H | H | 23 |
| [1] | 4.95 | F | H | F | H | OCH_3_ | H | H | 24 |
| [1] | 4.73 | Cl | H | Cl | H | OCH_3_ | OCH_3_ | H | 25 |
| [1] | 4.30 | N(CH_3_)_2_ | H | H | H | OCH_3_ | OCH_3_ | H | 26 |
| [1] | 4.81 | H | H | Cl | H | OCH_3_ | OCH_3_ | H | 27 |
| [1] | 4.26 | OCH_3_ | H | H | H | OCH_3_ | OCH_3_ | H | 28 |
| [1] | 4.18 | OCH_3_ | H | OCH_3_ | H | OCH_3_ | OCH_3_ | H | 29 |
| [1] | 4.45 | H | OCH_3_ | OCH_3_ | H | OCH_3_ | OCH_3_ | H | 30 |
| [1] | 4.86 | H | H | Br | H | OCH_3_ | OCH_3_ | H | 31 |

|  | | | | | | |
| --- | --- | --- | --- | --- | --- | --- |
| No. | R_1_ | R_2_ | R_3_ | R_4_ | pIC_50_ exp. | Reference |
| 32 | H | H | H | H | 4.31 | [1] |
| 33 | CH_3_ | H | H | H | 4.07 | [1] |
| 34 | OCH_3_ | H | H | H | 3.81 | [1] |
| 35 | H | H | H | Cl | 4.26 | [1] |
| 36 | OH | H | H | Cl | 4.22 | [1] |
| 37 | H | H | CH_3_ | H | 4.07 | [1] |
| 38 | OH | H | CH_3_ | H | 4.29 | [1] |
| 39 | CH_3_ | H | CH_3_ | H | 4.16 | [1] |
| 40 | OCH_3_ | H | CH_3_ | H | 4.20 | [1] |
| 41 | CH_3_ | OCH_3_ | H | H | 3.93 | [1] |
| 42 | H | N(CH_3_)_2_ | H | H | 3.59 | [1] |
| 43 | CH_3_ | N(CH_3_)_2_ | H | H | 3.58 | [1] |
| 44 | H | H | H | OH | 4.05 | [1] |
| 45 | CH_3_ | H | H | OH | 4.26 | [1] |
| 46 | OCH_3_ | H | H | OH | 4.35 | [1] |
| 47 | H | H | Cl | H | 4.49 | [1] |

|  | | | | | | |
| --- | --- | --- | --- | --- | --- | --- |
| No. | R_1_ | R_2_ | R_3_ | R_4_ | pIC50 exp. | Reference |
| 48 | OH | H | H | OC_2_H_5_ | 4.36 | [1] |
| 49 outlier | OC_2_H_5_ | H | H | OC_2_H_5_ | 5.50 | [1] |
| 50 | OH | H | H | OC_3_H_7_ | 3.90 | [1] |
| 51 | OH | H | H | OCH(CH_3_)_2_ | 4.52 | [1] |
| 52 | OH | H | H | OC_4_H_9_ | 4.15 | [1] |
| 53 | OH | H | H | OCH_2_CH(CH_3_)_2_ | 4.51 | [1] |
| 54 | OH | H | H | OC_5_H_11_ | 4.06 | [1] |
| 55 | OH | H | H | OCH_3_ | 4.15 | [1] |
| 56 outlier | OC_2_H_5_ | H | H | OCH_3_ | 7.00 | [1] |
| 57 | OC_3_H_7_ | H | H | OCH_3_ | 4.06 | [1] |
| 58 | OC_4_H_9_ | H | H | OCH_3_ | 4.21 | [1] |
| 59 | OH | OH | OCH_3_ | H | 4.27 | [1] |
| 60 | OH | OH | OH | H | 4.45 | [1] |

|  | | | | | |
| --- | --- | --- | --- | --- | --- |
| No. | R_1_ | R_2_ | R_3_ | pIC_50_ exp. | Reference |
| 61 | H | H | H | 5.82 | [1] |
| 62 | H | H | F | 4.92 | [1] |
| 63 | H | H | Cl | 5.22 | [1] |
| 64 | H | H | NO | 5.06 | [1] |
| 65 | H | H | OCH_3_ | 5.47 | [1] |
| 66 | H | H | OCF_3_ | 5.96 | [1] |
| 67 | H | H | CF_3_ | 5.43 | [1] |
| 68 | H | H | CH_3_ | 5.68 | [1] |
| 69 | H | H | OC_2_H_5_ | 5.62 | [1] |
| 70 | CH_3_ | H | H | 5.22 | [1] |
| 71 | H | CH_3_ | CH_3_ | 5.23 | [1] |

|  | | | |
| --- | --- | --- | --- |
| No. | R | pIC_50_ exp. | Reference |
| 72 | H | 5.08 | [1] |
| 73 | CH_3_ | 5.02 | [1] |

|  | | |
| --- | --- | --- |
| No. | pIC_50_ exp. | Reference |
| 74 outlier | 5.72 | [1] |

|  | | |
| --- | --- | --- |
| No. | pIC_50_ exp. | Reference |
| 75 | 4.64 | [1] |

|  | | |
| --- | --- | --- |
| No. | pIC_50_ exp. | Reference |
| 76 | 4.96 | [1] |

|  | | |
| --- | --- | --- |
| No. | pIC_50_ exp. | Reference |
| 77 | 4.94 | [1] |

|  | | |
| --- | --- | --- |
| No. | pIC_50_ exp. | Reference |
| 78 | 4.71 | [1] |

|  | | |
| --- | --- | --- |
| No. | pIC_50_ exp. | Reference |
| 79 | 4.79 | [1] |

|  | | | | | | |
| --- | --- | --- | --- | --- | --- | --- |
| No. | R_1_ | R_2_ | X | Z | pIC_50_ exp. | Reference |
| 80^t^ | H | H | O | - | 4.61 | [1] |
| 81 |  | - |  | O | 4.67 | [1] |
| 82 | H | H | S | - | 4.88 | [1] |
| 83 |  | - |  | S | 5.28 | [1] |
| 84 | CH_3_ | H | O | - | 4.37 | [1] |
| 85 outlier | CH_3_ | H | S |  | 5.75 | [1] |
| 86 | H | CH_3_ | S |  | 4.71 | [1] |
| 87 | Br | H | O |  | 4.82 | [1] |

|  | | |
| --- | --- | --- |
| No. | pIC_50_ exp. | Reference |
| 88 | 4.58 | [1] |

|  | | | | | |
| --- | --- | --- | --- | --- | --- |
| No. | R_1_ | R_2_ | R_3_ | pIC_50_ exp. | Reference |
| 89 | H | CH_3_ | H | 4.79 | [1] |
| 90 | H | OCH_3_ | H | 4.64 | [1] |
| 91 | OCH_3_ | OCH_3_ | OCH_3_ | 4.62 | [1] |

|  | | | | | | |
| --- | --- | --- | --- | --- | --- | --- |
| No. | R_1_ | R_2_ | R_3_ | R_4_ | pIC_50_ exp. | Reference |
| 92 | H | OCH_3_ | OCH_3_ | OCH_3_ | 4.82 | [1] |
| 93 | OCH_3_ | OCH_3_ | H | H | 4.92 | [1] |
| 94 | OCH_3_ | H | OCH_3_ | H | 4.79 | [1] |
| 95 | OCH_3_ | NO | H | H | 5.06 | [1] |
| 96 | OCH_3_ | OCH_3_ | OCH_3_ | OCH_3_ | 4.92 | [1] |
| 97 | OCH_3_ | H | F | H | 4.93 | [1] |

|  | | | | | | |
| --- | --- | --- | --- | --- | --- | --- |
| No. | R_1_ | R_2_ | R_3_ | R_4_ | pIC_50_ exp. | Reference |
| 98 | H | OCH_3_ | OCH_3_ | OCH_3_ | 4.68 | [1] |
| 99 | OCH_3_ | OCH_3_ | OCH_3_ | OCH_3_ | 5.19 | [1] |

|  | | | | | | |
| --- | --- | --- | --- | --- | --- | --- |
| No. | R1 | R_2_ | R_3_ | R_4_ | pIC_50_ exp. | Reference |

| 100 | H | OCH_3_ | OCH_3_ | OCH_3_ | 4.91 | [1] |
| --- | --- | --- | --- | --- | --- | --- |
| 101 | OCH_3_ | OCH_3_ | H | H | 5.09 | [1] |
| 102 | OCH_3_ | H | OCH_3_ | H | 4.77 | [1] |
| 103 | OCH_3_ | NO | H | H | 5.08 | [1] |
| 104 | OCH_3_ | OCH_3_ | OCH_3_ | OCH_3_ | 5.02 | [1] |
| 105^t^ | OCH_3_ | H | F | H | 4.67 | [1] |

|  | | | | | | |
| --- | --- | --- | --- | --- | --- | --- |
| No. | R_1_ | R_2_ | R_3_ | R_4_ | pIC_50_ exp. | Reference |
| 106 | H | OCH_3_ | OCH_3_ | OCH_3_ | 4.81 | [1] |
| 107 | OCH_3_ | OCH_3_ | OCH_3_ | OCH_3_ | 5.22 | [1] |

|  | | | |
| --- | --- | --- | --- |
| No. | R_1_ | pIC_50_ exp. | Reference |
| 108 | CH_3_ | 4.91 | [1] |
| 109 | Ph | 5.11 | [1] |

|  | | | |
| --- | --- | --- | --- |
| No. | R_1_ | pIC_50_ exp. | Reference |
| 110 | CH_3_ | 4.93 | [1] |
| 111 | Ph | 5.05 | [1] |

|  | | | | | |
| --- | --- | --- | --- | --- | --- |
| No. | R_1_ | R_2_ | R_3_ | pIC_50_ exp. | Reference |
| 112 outlier | H | H | H | 5.63 | [1] |
| 113 | H | F | H | 5.15 | [1] |
| 114 | H | Cl | H | 4.94 | [1] |
| 115 | H | Br | H | 4.72 | [1] |
| 116 | H | OCH_3_ | H | 4.75 | [1] |
| 117 outlier | H | OCF_3_ | H | 6.43 | [1] |
| 118 | H | NO | H | 4.74 | [1] |
| 119 | Cl | Cl | H | 4.68 | [1] |
| 120 | OCH_3_ | OCH_3_ | OCH_3_ | 5.08 | [1] |

|  | | | |
| --- | --- | --- | --- |
| No. | R_1_ | pIC_50_ exp. | Reference |
| 121 | 4-(Dimethylamino)phenyl | 5.73 | [1] |
| 122 | Naphthalen-2-yl | 5.35 | [1] |
| 123 outlier | 4-(Diethylamino)phenyl | 6.26 | [1] |
| 124 | 4-(Pyrrolidin-1-yl)phenyl | 5.08 | [1] |
| 125 | 4-(1H-Imidazol-1-yl)phenyl | 5.18 | [1] |
| 126 | Quinolin-2-yl | 5.42 | [1] |
| 127 | 4-Nitrophenyl | 5.43 | [1] |
| 128 | 3-Nitrophenyl | 5.37 | [1] |
| 129 | 3-(Trifluoromethyl)phenyl | 5.04 | [1] |
| 130 | Pyridin-2-yl | 5.67 | [1] |
| 131 | 3.4-Dichlorophenyl | 5.12 | [1] |
| 132 | 2-(4-Chloro)phenyl | 5.30 | [1] |

|  | | | |
| --- | --- | --- | --- |
| No. | R_1_ | pIC_50_ exp. | Reference |
| 133 | 2.4-Difluorophenyl | 6.03 | [1] |
| 134 | 3.4.5-Trimethoxyphenyl | 6.62 | [1] |
| 135 | 4-Triflouromethoxyphenyl | 6.44 | [1] |
| 136 | 3-Triflouromethoxyphenyl | 6.59 | [1] |
| 137 | 4-Fluorophenyl | 6.16 | [1] |
| 138 | 4-Chlorophenyl | 6.30 | [1] |
| 139 | 2.6-Dichlorophenyl | 6.85 | [1] |
| 140 | 3-Methoxyphenyl | 6.14 | [1] |
| 141 | Phenyl | 6.19 | [1] |
| 142 | 2-Furyl | 5.64 | [1] |
| 143 | 2-Thienyl | 5.80 | [1] |
| 144 | 1.3-Benzodioxo-5-yl | 5.92 | [1] |

|  | | | |
| --- | --- | --- | --- |
| No. | R_1_ | pIC_50_ exp. | Reference |
| 145 | 2.4-Difluorophenyl | 5.59 | [1] |
| 146 | 3.4.5-Trimethoxyphenyl | 6.16 | [1] |
| 147 | 4-Triflouromethoxyphenyl | 6.41 | [1] |
| 148 | 3-Triflouromethoxyphenyl | 6.62 | [1] |
| 149 | 4-Fluorophenyl | 6.12 | [1] |
| 150 | 4-Chlorophenyl | 6.17 | [1] |
| 151 | 2.6-Dichlorophenyl | 6.30 | [1] |
| 152 | 3-Methoxyphenyl | 6.17 | [1] |
| 153 | Phenyl | 6.13 | [1] |
| 154 | 2-Furyl | 5.85 | [1] |
| 155 | 2-Thienyl | 5.68 | [1] |
| 156 | 1.3-Benzodioxo-5-yl | 5.77 | [1] |

| 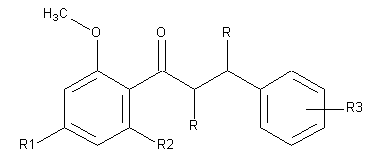 | | | | | | |
| --- | --- | --- | --- | --- | --- | --- |
| No. | R | R_1_ | R_2_ | R_3_ | pIC_50_ exp. | Reference |
| 157 | H | OCH_3_ | OH | 3, 4 OCH_2_OH | 3.84 | [1] |
| 158 | Double bond | OCH_3_ | OH | 3 OCH_3_ | 4.34 | [1] |
| 159 | Double bond | OH | OH | 3 OCH_3_ | 4.67 | [1] |
| 160 | Double bond | OH | OH | 3, 4 OCH_2_OH | 4.68 | [1] |
| 161 | Double bond | OCH_2_CH=CH_2_ | OCH_2_CH=CH_2_ | 3 OCH_3_ | 4.55 | [1] |
| 192 | Double bond | OCH_2_CH=CH_2_ | OCH_2_CH=CH_2_ | 3, 4 OCH_2_OH | 4.62 | [1] |

|  | | | | | | | | |
| --- | --- | --- | --- | --- | --- | --- | --- | --- |
| No. | R^1^ | R^2^ | R^3^ | R^4^ | R^5^ | R^6^ | In Vitro IC_50_(µM) | Reference |
| 193 | OCH_3_ | OCH_3_ | OCH_3_ | OCH_3_ | OCH_3_ | OCH_3_ | 4.64 | [2] |
| 162 | OCH_3_ | OCH_3_ | OCH_3_ | H | NO_2_ | H | 4.32 | [2] |
| 163 | OCH_3_ | OCH_3_ | OCH_3_ | H | NH_2_ | H | 4.26 | [2] |
| 164 | H | F | OCH_3_ | H | OCH_3_ | H | 4.22 | [2] |

|  | | | | | | | |
| --- | --- | --- | --- | --- | --- | --- | --- |
| No. | R | R_1_ | R_2_ | R_3_ | R_4_ | IC_50_(µM) | Reference |
| 165 | OMe | H | OMe | OMe | OMe | 4.69 | [3] |
| 166 | OMe | H | OMe | H | OMe | 4.91 | [3] |
| 167 | OMe | H | OMe | OMe | H | 4.59 | [3] |
| 168 | OMe | H | OH | OMe | H | 4.92 | [3] |
| 169 | OMe | Br | OMe | OMe | OMe | 4.46 | [3] |
| 170 | OEt | H | OMe | OMe | OMe | 4.42 | [3] |
| 171 | OEt | H | OMe | H | OMe | 4.54 | [3] |
| 172 | OEt | H | OMe | OMe | H | 4.47 | [3] |
| 173 | OEt | H | OH | OMe | H | 4.93 | [3] |
| 174 | OEt | H | H | OMe | H | 4.91 | [3] |
| 175 | OEt | Br | OMe | OMe | OMe | 4.69 | [3] |
| 176 | Me | H | OMe | OMe | OMe | 4.74 | [3] |
| 177 | Me | H | OMe | H | OMe | 4.56 | [3] |
| 178 | Me | H | OMe | OMe | H | 5.02 | [3] |
| 179 | Me | H | H | OMe | H | 4.97 | [3] |
| 180 | Me | Br | OMe | OMe | OMe | 4.67 | [3] |
| 181 | H | H | OMe | OMe | OMe | 4.61 | [3] |
| 182 | H | H | OMe | H | OMe | 4.61 | [3] |
| 183 | H | H | OMe | OMe | H | 5.35 | [3] |
| 184 | H | H | OH | OMe | H | 5.15 | [3] |
| 185 | H | H | H | OMe | H | 5.66 | [3] |
| 186 | H | Br | OMe | OMe | OMe | 5.04 | [3] |
| 187 | F | H | OMe | OMe | OMe | 5.13 | [3] |
| 188 | F | H | OMe | H | OMe | 4.97 | [3] |
| 189 | F | H | OMe | OMe | H | 4.81 | [3] |
| 190 | F | H | H | OMe | H | 5.32 | [3] |
| 191 | F | Br | OMe | OMe | OMe | 5.09 | [3] |

1. Rybka M, Mercader AG, Castro EA: **Predictive QSAR study of chalcone derivatives cytotoxicity activity against HT-29 human colon adenocarcinoma cell lines**. *Chemometrics and Intelligent laboratory systems* 2014, **132**:18-29.

2. Shankaraiah N, Siraj K, Nekkanti S, Srinivasulu V, Sharma P, Senwar KR, Sathish M, Vishnuvardhan M, Ramakrishna S, Jadala C: **DNA-binding affinity and anticancer activity of β-carboline–chalcone conjugates as potential DNA intercalators: Molecular modelling and synthesis**. *Bioorganic chemistry* 2015, **59**:130-139.

3. Sultana F, Bonam SR, Reddy VG, Nayak VL, Akunuri R, Routhu SR, Alarifi A, Halmuthur MSK, Kamal A: **Synthesis of benzo [d] imidazo [2, 1-b] thiazole-chalcone conjugates as microtubule targeting and apoptosis inducing agents**. *Bioorganic chemistry* 2018, **76**:1-12.

**Reference**
